# A novel prognostic scoring model based on copper homeostasis and cuproptosis which indicates changes in tumor microenvironment and affects treatment response

**DOI:** 10.3389/fphar.2023.1101749

**Published:** 2023-02-24

**Authors:** Yun-Long Ma, Ya-Fei Yang, Han-Chao Wang, Chun-Cheng Yang, Lun-Jie Yan, Zi-Niu Ding, Bao-Wen Tian, Hui Liu, Jun-Shuai Xue, Cheng-Long Han, Si-Yu Tan, Jian-Guo Hong, Yu-Chuan Yan, Xin-Cheng Mao, Dong-Xu Wang, Tao Li

**Affiliations:** ^1^ Department of General Surgery, Qilu Hospital, Shandong University, Jinan, China; ^2^ Institute for Financial Studies, Shandong University, Jinan, China; ^3^ Department of hepatobiliary surgery, The Second Hospital of Shandong University, Jinan, China

**Keywords:** copper homeostasis, cuproptosis, hepatocellular carcinoma, prognostic model, tumor microenvironment, immunocytes, intercellular communication

## Abstract

**Background:** Intracellular copper homeostasis requires a complex system. It has shown considerable prospects for intervening in the tumor microenvironment (TME) by regulating copper homeostasis and provoking cuproptosis. Their relationship with hepatocellular carcinoma (HCC) remains elusive.

**Methods:** In TCGA and ICGC datasets, LASSO and multivariate Cox regression were applied to obtain the signature on the basis of genes associated with copper homeostasis and cuproptosis. Bioinformatic tools were utilized to reveal if the signature was correlated with HCC characteristics. Single-cell RNA sequencing data analysis identified differences in tumor and T cells’ pathway activity and intercellular communication of immune-related cells. Real-time qPCR analysis was conducted to measure the genes’ expression in HCC and adjacent normal tissue from 21 patients. CCK8 assay, scratch assay, transwell, and colony formation were conducted to reveal the effect of genes on *in vitro* cell proliferation, invasion, migration, and colony formation.

**Results:** We constructed a five-gene scoring system in relation to copper homeostasis and cuproptosis. The high-risk score indicated poor clinical prognosis, enhanced tumor malignancy, and immune-suppressive tumor microenvironment. The T cell activity was markedly reduced in high-risk single-cell samples. The high-risk HCC patients had a better expectation of ICB response and reactivity to anti-PD-1 therapy. A total of 156 drugs were identified as potential signature-related drugs for HCC treatment, and most were sensitive to high-risk patients. Novel ligand-receptor pairs such as FASLG, CCL, CD40, IL2, and IFN-Ⅱ signaling pathways were revealed as cellular communication bridges, which may cause differences in TME and immune function. All crucial genes were differentially expressed between HCC and paired adjacent normal tissue. Model-constructed genes affected the phosphorylation of mTOR and AKT in both Huh7 and Hep3B cells. Knockdown of ZCRB1 impaired the proliferation, invasion, migration, and colony formation in HCC cell lines.

**Conclusion:** We obtained a prognostic scoring system to forecast the TME changes and assist in choosing therapy strategies for HCC patients. In this study, we combined copper homeostasis and cuproptosis to show the overall potential risk of copper-related biological processes in HCC for the first time.

## 1 Introduction

As an indispensable human body element, copper participates in various physiological and metabolic functions, including coagulation, oxidative metabolism, and hormone production (Bhattacharjee et al., 2017). There are inherent complex mechanisms in cells to maintain copper homeostasis. Copper homeostasis disorders involve a wide range of diseases, including degenerative neurological diseases (Bisaglia and Bubacco, 2020), metabolic diseases (Lowe et al., 2017), cardio-cerebrovascular diseases (Fukai et al., 2018), and tumors (Oliveri, 2022). The elevated copper level has been found in various solid tumors, promoting proliferation, invasion, migration, and angiogenesis (Oliveri, 2022). Excess copper caused by copper homeostasis disorder such as transporter mutation leads to programmed cell death, which was recently identified as cuproptosis (Tsvetkov et al., 2022). Cuproptosis is induced *via* copper-dependent protein fatty acylation, accompanied by tricarboxylic acid cycle changes, and influenced by mitochondrial function (Tsvetkov et al., 2022). Copper homeostasis is not only related to the drug resistance of traditional chemotherapeutic drugs but also can affect specific immune checkpoints and change the anti-tumor immune response (da Silva et al., 2022; Voli et al., 2020). Given the vital role of copper in cancer, copper ion carriers and copper complexes have been developed as anticancer drugs (Chen et al., 2006; Cen et al., 2004; O'Day et al., 2009; O'Day et al., 2013; Tsang et al., 2020). Still, the metabolic heterogeneity of different cancers is the main obstacle to their application. To achieve a more stable and reliable anticancer effect by affecting the copper homeostasis of tumor cells, the corresponding receptors of specific types of tumor cells should be targeted (da Silva et al., 2022). As key players in this novel cell death form, the genes related to copper homeostasis and cuproptosis possibly be promising cancer therapy targets. The specific mechanism of cuproptosis was covered; nevertheless, for further targeted drug development and clinical application, understanding different targets of copper homeostasis and cuproptosis in various tumors is still far from sufficient.

Although there are a variety of measures for diagnosis and treatment, mortality and prognosis are still poor for HCC because of a wide range of predisposing factors and unobvious early clinical manifestations (Hartke et al., 2017). Compared with mature traditional therapy, non-invasive diagnosis and targeted therapy are still challenging. Recently, patients’ prognosis and life quality have been improved by systemic therapies (Llovet et al., 2021). The disorder of copper homeostasis can cause cuproptosis, which has great potential in developing new therapies for HCC. Copper content is closely linked to liver cirrhosis and HCC (Zhang et al., 1994). Ionizing radiation can increase the radiation resistance caused by intracellular copper and inhibit ferroptosis and the degradation of HIF1α (Yang et al., 2022). Copper-binding enzyme LOXL4 causes the immunosuppressive phenotype of macrophages and promotes the progression of HCC (Tan et al., 2021). Given the critical role of copper in HCC, new copper complexes for specific targets have been developed. A new copper complex can induce cell senescence by inhibiting methionine cycle metabolism, which depends on mitochondrial carrier protein (Jin et al., 2020). Another targeted nanoparticle containing copper complex effectively reduces the growth of mice’s HCC (Xu et al., 2020). The evidence above suggests that genes related to copper homeostasis and cuproptosis have remarkable research prospects in expanding systemic therapy and improving patient prognosis in clinical application.

This study developed a novel prognostic scoring system that incorporates genes related to copper homeostasis and cuproptosis to predict the clinical outcome of HCC patients. To demonstrate the predictive value of the signature, we explored the underlying mechanisms based on bulk and single-cell RNA sequencing data. Novel receptor-ligand pairs were proposed to help understand tumor-immune cell interactions and explain the differences in TME related to the signature. Finally, potential targeted and chemotherapeutic drugs were predicted for different scoring samples. Our predictive model showed great potential in identifying the risk of copper-related physiological processes and assisting in the therapy of HCC patients.

## 2 Materials and methods

### 2.1 Acquisition of multiomics data

The following bulk RNA-sequencing expression profiles and corresponding clinical data were downloaded from the TCGA database (https://portal.gdc.com *n* = 377). Raw sequencing reads were aligned using the STAR aligner and expressed as fragments per million mapped reads (FPKM). Gene expression profiles were standardized using R (https://www.r-project.org/). Only patients with complete clinical information related to the analysis were retained. Training and testing groups were randomly assigned in a ratio of 1:1 among the patients. To establish an independent validation cohort, Clinical pathology and RNA-Seq mRNA expression data were obtained for 232 samples from the ICGC portal (https://dcc.icgc.org/projects/LIRI-JP). The UCSC Xena server was used to retrieve somatic mutations and methylation data for HCC (https://xenabrowser.net/). The GEO database was used to download data for single-cell RNA sequencing of primary HCC tissues (GSE149614, *n* = 10). “Seurat” and “NormalizeData” R packages were used for the standardization of the single-cell RNA-Seq data. “FingVariableGenes” R package was used for the identification of the top 3,000 highly variable genes. The determination of cell types was as shown in Supplementary Figure S1A (Malignant cell markers-GPC3, CD24, MDK, KRT18; Meyloid cell markers-CD68, AIF1, C1QA, TPSAB1; T cell markers-CD3D, CD3E, CD2; B cell markers-MZB1, MS4A1, CD79A; Fibroblast cell markers-COL1A2, COL3A1, ACTA2; Endothelial cell markers-FLT1, RAMP2, PLVAP).

### 2.2 Identification of genes related to copper homeostasis and cuproptosis

25 genes (SLC31A1, SLC31A2, ATOX1, PDHB, COX11, COX17, PDHA1, NLRP3, NFE2L2, CCS, MTF1, LIPT2, LIPT1, LIAS, GLS, GCSH, FDX1, DLST, DLD, DLAT, DBT, CDKN2A, ATP7B, ATP7A, SCO1) directly involved in copper death and copper homeostasis processes were obtained from previous studies (Bian et al., 2022; da Silva et al., 2022; Inesi, 2017; Tsvetkov et al., 2022). An analysis of the differential expression of these genes was conducted in HCC. To screen related genes, Pearson correlation analysis was conducted (correlation coefficient>0.4, *p* < 0.001). Qualified genes were associated with cuproptosis or copper homeostasis.

### 2.3 Development of the signature related to copper homeostasis and cuproptosis

With R package “glmnet,” genes associated with copper homeostasis and cuproptosis were screened using univariate cox regression. Then the least absolute shrinkage and selection operator (LASSO) Cox regression and multivariate Cox regression models were used to creating the copper metabolism and cuproptosis gene signature in the training cohort. Gene expression values and coefficients of crucial genes were multiplied to determine the score of each sample. The median value of the score determined high-risk and low-risk groups. ROC curves analysis and Kaplan-Meier survival analysis were conducted to evaluate the signature. Independent prognostic analysis was conducted to determine if the risk score affected survival in patients with HCC. A stratified clinical examination was performed according to the patient’s clinical pathological characteristics (age, gender, grading, staging). The “ggDCA” R package was used to analyze different diagnostic models.

### 2.4 Nomogram development and validation

Multivariate Cox regression results were used to develop the Nomogram model. The final model was chosen using the Akaike information criterion (AIC) as a backward selection criterion (Harrell et al., 1996). Nomogram validation was conducted using a calibration curve generated *via* regression analysis. The nomogram was developed following the nomogram guide (Iasonos et al., 2008).

### 2.5 Functional enrichment and genetic alterations analysis

KEGG and GO analyses were performed using the R package “clusterProfiler” (Yu et al., 2012). The genetic variation between groups of Risk Scores was analyzed using R package “Maftools.” The ssGSEA score was calculated with R package “GSVA,” which was also used for functional enrichment analysis in malignant cells and T cells of single-cell RNA-Seq data. MATH score was used to evaluate tumor heterogeneity (Mroz and Rocco, 2013).

### 2.6 Drug sensitivity prediction

Drug sensitivity in cancer was predicted using the Genomics of Drug Sensitivity in Cancer database (GDSC: https://www.cancerxgene.org). “pRRophetic” R package was used to calculate half maximal inhibitory concentration (IC50) (Geeleher et al., 2014).

### 2.7 Immune profile analysis and cell communication

“Immunedeconv” R package was applied to evaluate the immune score (Sturm et al., 2020). VEGFB, TNFSF4, TNFRSF4, TNFRSF18, TIGIT, TGFB1, SELP, PDCD1, LAG3, IL1A, IL12A, IDO1, HMGB1, HAVCR2, EDNRB, CTLA4, CD276, CD274, CTLA4, BTLA, and ARG1 were chosen as immune checkpoints. The TIDE procedure was combined with subclass mapping and immunophenoscore (IPS) to calculate potential ICB responses (Kim et al., 2008). The IPS of HCC patients included in the analysis came from the TCIA database (https://www.tcia.at/home).

‘‘Celltalker’’ R package was applied to analyze crosstalk between malignant cells and immunocytes based on the single-cell RNA-Seq data.

### 2.8 Human tissues

Surgically resected HCC and normal adjacent tissue samples were obtained from twenty-one HCC patients at the Qilu Hospital of Shandong University (Jinan, China) and stored in liquid nitrogen. All HCC samples were confirmed through clinicopathological features. The hospital’s ethical committee approved the study, and each patient signed a written informed consent form.

### 2.9 qRT-PCR, Western blot, and immunohistochemistry

The cells were washed with PBS and lysed in RIPA buffer (Beyotime, CN) containing phosphatase inhibitors and protease inhibitors (Beyotime, CN) at the indicated time points. The BCA Protein Assay kit (Beyotime, CN) was used to determine protein lysate concentration. After centrifuging at 12,000×g for 15 min, the supernatant was mixed with the 5×SDS-PAGE loading buffer (Beyotime, CN), and boiled at 95°C for 5 min. A standard Western blot procedure was then followed. 0.2 um PVDF membrane was obtained from Thermo Fisher Scientific (Thermo Fisher Scientific, United States). Enhanced chemiluminescence was obtained from Thermo Fisher Scientific (Thermo Fisher Scientific, United States). Antibodies against AKT, p-AKT, mTOR, p-mTOR and GAPDH were obtained from Cell Signaling Technology (Cell Signaling Technology, CN).

Trizol reagent (Thermo Fisher Scientific, United States) was used to prepare total RNA from tissues or cells. PrimeScript™ RT Master Mix (Takara Bio, JP) was used for reverse transcription. qRT-PCR analysis was performed with the CFX Connect system (Bio-Rad, United States) and CharmQ SYBR qPCR Master Mix (Takara, Japan). Supplementary Table S3 lists the primers used in this study**.**

In accordance with standard protocols, immunohistochemistry was performed on HCC and adjacent normal tissue. Antibody against ZCRB1 was obtained from Thermo Fisher Scientific (Thermo Fisher Scientific, United States).

### 2.10 Cell lines and cell culture

Hep3B and Huh-7 cells were obtained from the Shanghai Cell Collection. DMEM with 10% FBS and 1% Penicillin-Streptomycin was used to culture the cells at 37°C with 5% CO2.

### 2.11 Cell transfection and Cell Counting Kit-8 assay

siCDKN2A, siDLAT, siGEMIN2, siZCRB1, and siKLF9 were obtained from Ribobio (CN). As directed by the manufacturer, JetPRIME® transfection kit (BIOFIL, CN) was used for transfection. After 24 h, total RNA was extracted for qRT-PCR.

Huh7 or Hep3B cells were inoculated into 96-well plates 24 h after transfection at a density of 1,000 cells per well. Each group was replicated five times. Cell Counting Kit-8 (Dojindo, JP) was used for the measurement of cell proliferation. As the culture progressed, absorbance values were measured after 0, 24, 48, and 72 h.

### 2.12 Migration, invasion, and colony formation assay

The scratch assay was applied to evaluate cell migration and repair. After reaching 90%–100% confluency in wells of culture plates, cells were exposed to serum-free medium for 6 h, and each cultured well was scraped with a pipette tip in the same specification. Cells were washed with PBS to remove fragments. Microscope images of the same positions were acquired in after 0 and 30 h. Based on the percentage of wound closure area, cell migration was determined.

Transwell migration and invasion assays were conducted to evaluate the ability of cell migration and invasion. 24-well transwell chambers (Corning, United States) were used in the assay. For the invasion assay, matrigel (Corning, United States) was applied to the upper ventricle surface of the basement membrane of the transwell chamber. The insert was filled with 30,000 cells suspended in 150 ul serum-free serum before the assay. In the lower chamber, 700 ul medium containing 12% fetal serum was added for chemotactic stimulation. Cells were cultured for 24 h for migration assays and 40 h for invasion assays. Then cotton swabs were used to remove cells from the surface of the membrane. Cultured cells were fixed with 100% methanol and stained with 0.1% crystal violet. Random visual fields of 3 different inserts were captured, and the number of cells was counted.

After inoculating 3000 cells per well, Huh7 and Hep3B cells were grown for 8 and 10 days respectively in 6 well plates in complete medium. Cultured cells were fixed with 100% methanol and stained with 0.1% crystal violet. Each well was counted for the number of colonies.

### 2.13 Statistical analysis

R packages and analysis methods were executed with R (version 4.0.3). Quantitative variables were evaluated using independent samples t-tests. The unpaired Wilcoxon rank sum test was applied for the gene difference significance. For categorical data, Chi-square tests were applied. The ROC curve and Kaplan-Meier model judged efficacy in predicting survival outcomes. The relationships between prognostic classification, survival outcomes, and other clinical parameters were revealed with the Cox proportional model. A *p*-value less than 0.05 indicates statistical significance. * means a *p*-value less than 0.05; ** means a *p*-value less than 0.01; *** means a *p*-value less than 0.001; **** means a *p*-value less than 0.0001. For multiple corrections, the Benjamini–Hochberg method was applied.

## 3 Results

### 3.1 Identification of genes related to copper homeostasis and cuproptosis and development of prognostic signature

**FIGURE 1:**
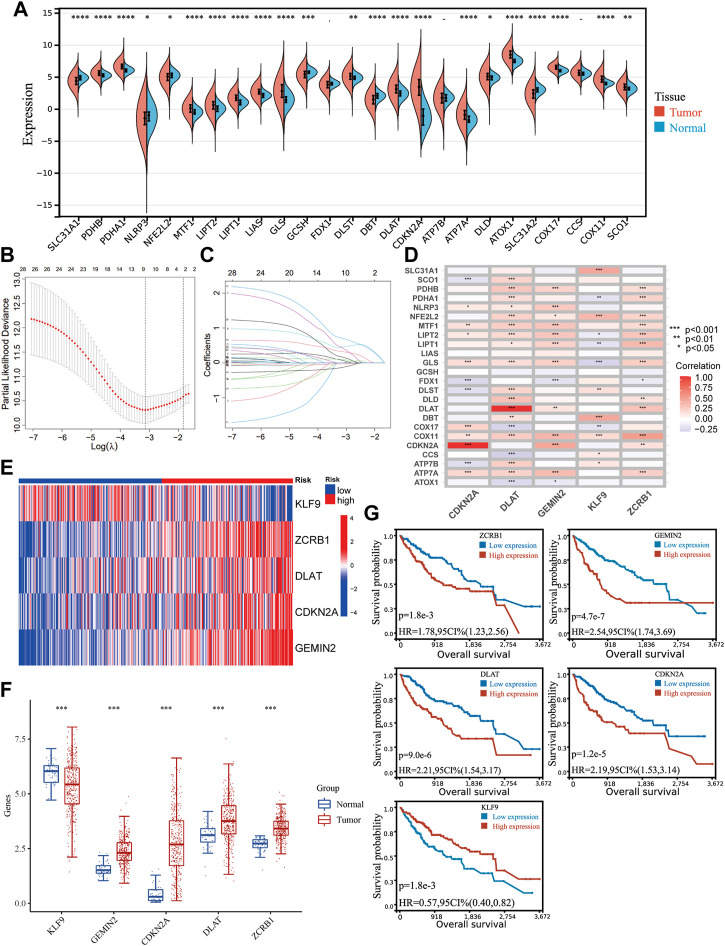
Identifying genes related to copper homeostasis and cuproptosis and development of the signature. **(A)** Expression of 25 genes in HCC and normal tissues. Unpaired Wilcoxon Rank Sum and Signed Rank Test was applied for difference significance analysis. **(B)** Ten-fold cross-validation of the LASSO Cox regression model’s tuning parameter (*λ*) selection. Based on the minimum criteria and the 1-SE criteria, vertical lines were drawn at the optimal values. **(C)** LASSO coefficient profiles of the prognostic genes. **(D)** The correlation between the five genes in the signature and the genes directly participating in copper homeostasis and cuproptosis. **(E)** The expression of the five hub genes in the TCGA cohort. **(F)** Differential expression of the hub genes between HCC and normal tissues. **(G)** Kaplan−Meier plots of CDKN2A, KLF9, DLAT, GEMIN2, and ZCRB1.

We sorted out 25 genes from previous studies that have been proven to participate in cuproptosis and the maintenance of copper homeostasis directly. Most genes (22/25) were differently expressed in HCC and normal tissues (Figure 1A). Given the crucial role of copper in cancer, the signature related to cuproptosis and copper homeostasis could assist in evaluating tumor microenvironment changes and other pathological processes in HCC induced by copper. A correlation analysis was carried out according to the coefficient, and 95 genes were screened.

Including the original set of genes and their related genes, one hundred twenty candidate genes were confirmed as genes related to copper homeostasis and cuproptosis [Supplementary Table S1 (S1)], which were input into a univariate COX analysis. A LASSO regression was conducted on the genes with prognostic significance. Five hub genes were obtained (CDKN2A, DLAT, KLF9, GEMIN2, ZCRB1) (Figures 1B, C). There is a strong correlation between hub genes and genes directly participating in copper homeostasis and cuproptosis (Figure 1D). The signature was developed with a multivariate Cox proportional model. Risk Score = −0.2629*KLF9+0.6633*ZCRB1 +0.3994*DLAT+ 0.2121* CDKN2A+0.7650*GEMIN2. Gene expression in the cohort was visualized using a heat map (Figure 1E). HCC and normal tissues expressed all five genes differently (Figure 1F). Kaplan-Meier survival analysis was used to verify their relationship with HCC prognosis (Figure 1G). Five hub genes were significantly different in expression between HCC and normal tissues, and their expression was correlated with prognosis.

### 3.2 Clinical prognostic validation of the signature

**FIGURE 2:**
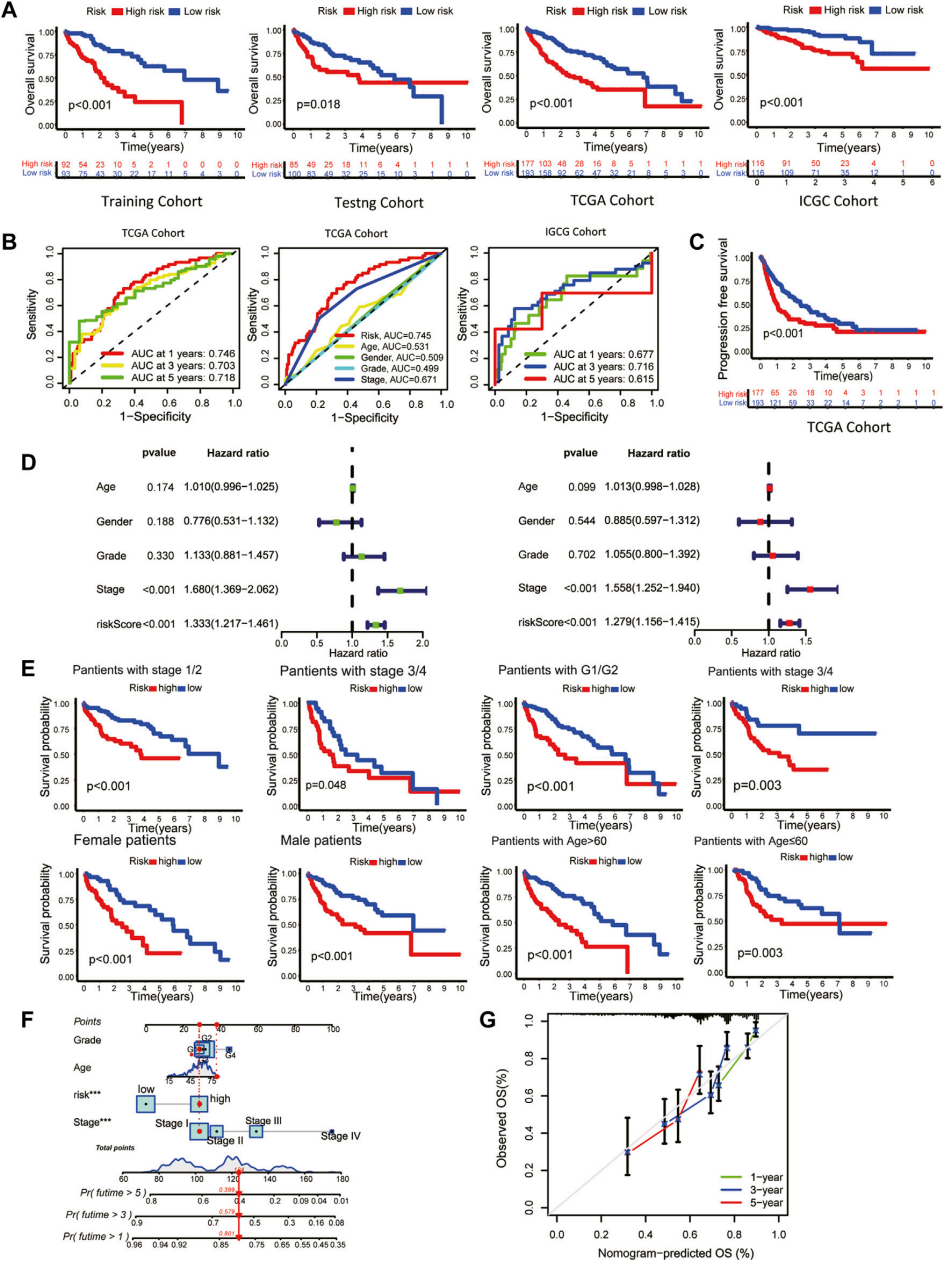
Clinical validation of the prognostic signature. **(A)** The Kaplan-Meier overall survival (OS) curves of the risk score in the training, testing, TCGA, and ICGC cohorts. **(B)** Receiver operating characteristic (ROC) curve based on the risk score and other clinicopathological features for predicting OS in HCC patients. **(C)** Kaplan-Meier Progression–free Survival (PFS) curve. **(D)** Univariate (left) and multivariate (right) Cox regression analyses. **(E)** Kaplan-Meier survival subgroup analysis stratified by clinical characteristics. Grouping criteria: age>60/≤60, gender, histological grade, TNM stage. **(F)** OS nomogram **(G)** Nomogram calibration for the OS nomogram.

The overall survival (OS) of high-risk patients was briefer in all cohorts (Figure 2A). In the TCGA cohort, 1-year, 3-year, and 5-year AUC values were 0.746, 0.703, and 0.718. Compared with clinical features, the risk score has higher prediction accuracy (Figure 2B). In addition, the Progression Free Survival (PFS) of high-risk patients in the TCGA cohort was also shorter (Figure 2C). Clinical characteristics and risk scores of HCC patients were analyzed by univariate and multivariate Cox regression, demonstrating that prognosis was independently predicted by the risk score (Figure 2D). The correlation between the risk score and pathological characteristics was examined. T stage, TNM stage, and histological grade were significantly correlated with risk score (Table 1). In stratified clinical analysis, there were significant differences in OS between high-risk and low-risk patients in all subgroups (Figure 2E). As an independent external validation set, the ICGC dataset was processed using the same methodology as the TCGA dataset. AUC, pathological characteristics analysis, and Kaplan-Meier analysis of the ICGC dataset once again demonstrated the prognostic value of the signature (Figures 2A, B) (Table 1). According to the above results, the signature was associated with HCC progression.

**TABLE 1:** The correlation between risk score and clinicopathological features of HCC patients in the TCGA and ICGC cohort.

	TCGA-LIHC cohort (*n* = 377)			LIRI-JP cohort (*n* = 232)		
	High risk	Low risk	*p*-Value	High risk	Low risk	*p*-Value
Age			0.08			0.425
≤60	86	91		22	28	
>60	89	102		94	88	
Gender			0.923			0.371
Female	58	62		34	27	
Male	117	131		82	89	
Child_pugh			0.788			NA
A	87	129		NA	NA	
B/C	10	12		NA	NA	
AFP			0.512			NA
≤300	60	58		NA	NA	
>300	45	54		NA	NA	
Fibrosis/Cirrhosis			0.108			NA
No	25	94		NA	NA	
Yes	24	50		NA	NA	
T_stage			0.020*			NA
T1	72	109		NA	NA	
T2	51	42		NA	NA	
T3	44	34		NA	NA	
T4	8	5		NA	NA	
N_stage			0.361			NA
N0	121	129		NA	NA	
N1	3	1		NA	NA	
M_stage			0.625			NA
M0	126	139		NA	NA	
M1	1	3		NA	NA	
TNM_stage			0.010*			<0.001*
I	67	104		13	23	
II	43	42		42	64	
III	49	34		45	26	
IV	1	4		16	3	
Histological_grade			<0.001*			NA
G1	16	39		NA	NA	
G2	75	102		NA	NA	
G3	73	46		NA	NA	
G4	9	3		NA	NA	

AFP, alpha-fetoprotein; TNM, cancer staging system.

As a means of facilitating the clinical application of prognostic signatures, a nomogram was constructed through the combination of traditional clinical information (age, tumor stage, tumor grade) and risk scores (Figure 2F). Based on the second-generation sequencing result of the patients, the overall survival can be estimated by combining pathological characteristics with the risk scores. The nomogram performed well at predicting according to the calibration curve (Figure 2G).

### 3.3 Distribution of model-constructing genes and risk score in UMAP

**FIGURE 3:**
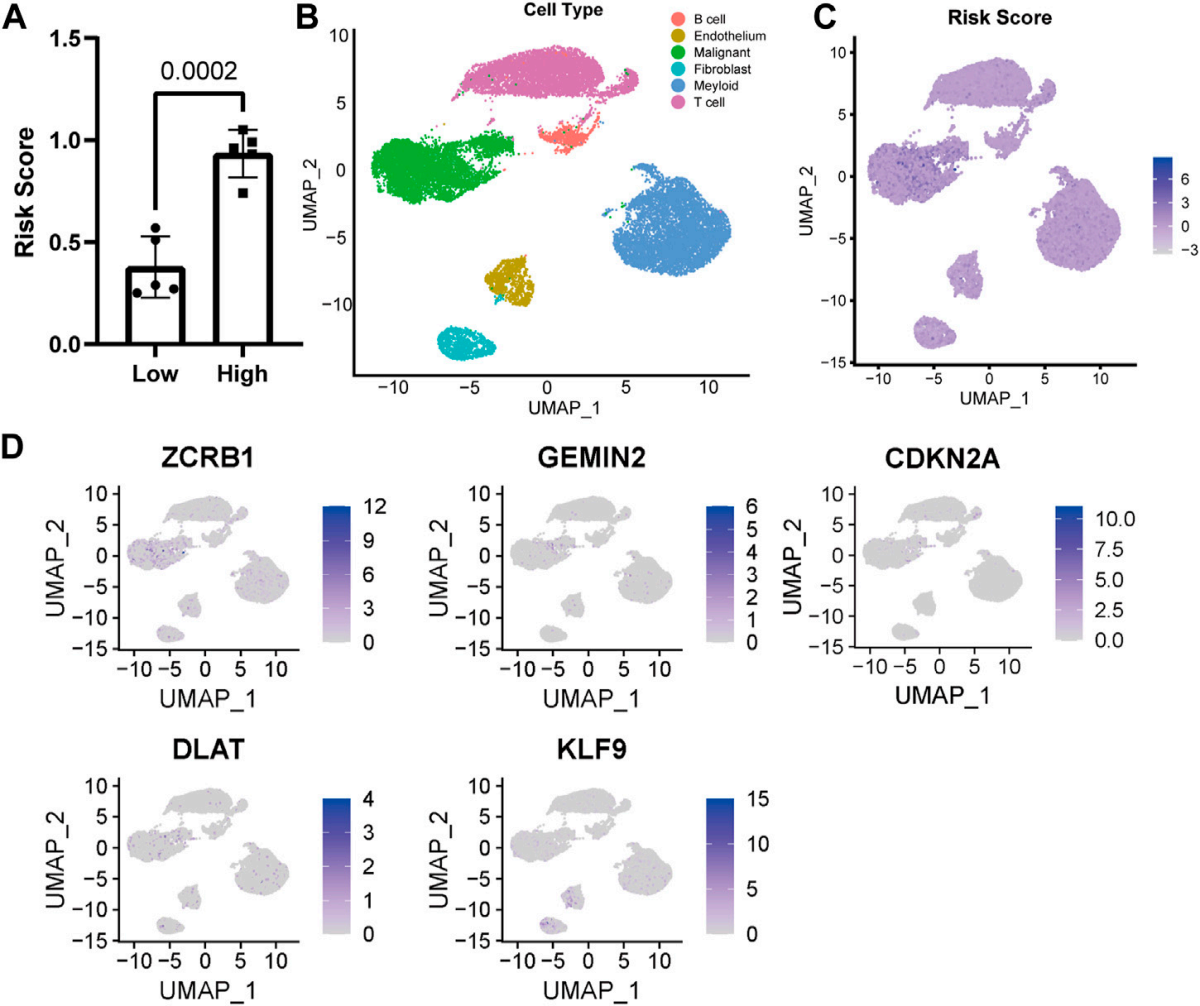
Distribution of model genes and risk score in UMAP based on single-cell sequencing data (GSE149614). **(A)** Comparison of risk scores of different samples. **(B)** UMAP of 23,590 cells from primary HCC tumors of ten HCC patients. **(C)** Distribution of risk score in UMAP. **(D)** Distribution of model construction genes in UMAP.

The risk score of ten single-cell sequencing samples was calculated according to the Cox proportional model above for further analysis [Supplementary Table S1 (S2)]. We divided the single-cell sequencing samples into high- and low-risk scoring groups, and there was a significant statistical difference between the two groups (Figure 3A). Given the excellent predictability of the risk score for clinical prognosis, we explored the distribution of genes participating in the risk model and the risk score distribution in the uniform manifold approximation and projection (UMAP) based on the single-cell RNA sequencing data. The cells with high-risk scores were mainly malignant (Figures 3B, C). All the genes involved in the model construction were expressed to a certain extent in malignant cells (Figure 3D). Different from other genes, increased expression of KLF9 is associated with a lower risk score and better prognosis for patients, while it’s mainly expressed in fibroblasts and endothelial cells (Figure 3D).

### 3.4 Functional enrichment analysis revealed risk score correlated with HCC malignant degree

**FIGURE 4:**
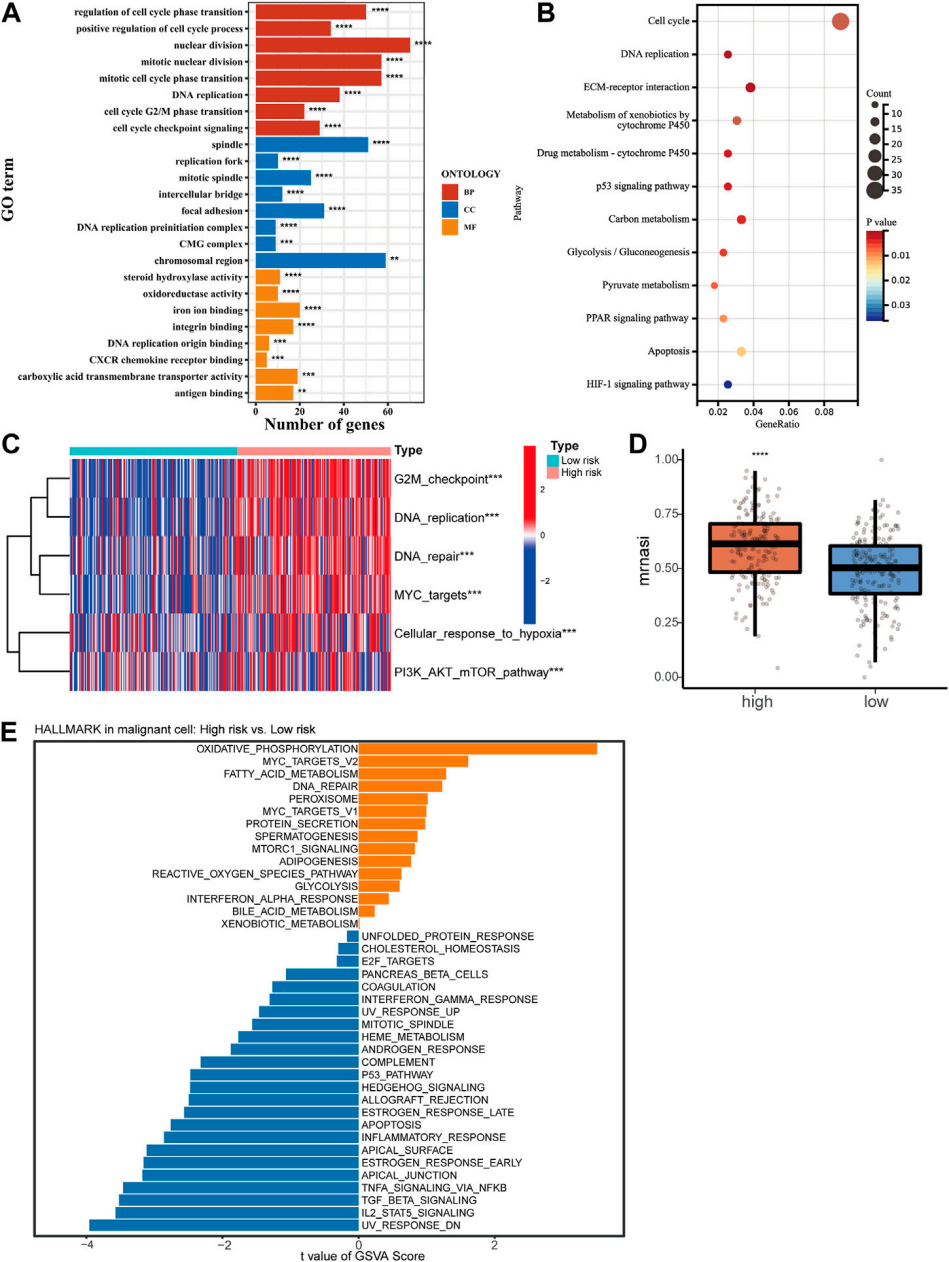
Functional enrichment analysis of high- and low-risk groups. GO analysis **(A)** and KEGG analysis **(B)** of DEGs between the high-risk and low-risk groups. **(C)** Heatmap of ssGSEA scores in proliferation-related pathways. **(D)** Correlation between mRNAsi score and risk score of the signature. **(E)** GSVA for malignant cells from single-cell RNA-Seq.

To reveal the potential mechanism causing the clinical characteristic in HCC patients with different risk scores, a cut-off of a *p*-value of 0.05 and a |FC| > 2 was used for screening differentially expressed genes (DEGs) between high- or low-risk groups [Supplementary Table S1 (S3)]. An analysis of GO and KEGG was then conducted. Results showed cell proliferation-related biological processes enriched mostly (Figures 4A, B). Then we collected a set of genes in tumor-related pathways and calculated the enrichment scores for every patient using the ssGSEA method. The high-risk group showed significant upregulation of proliferation and cell cycle pathways, including G2M checkpoint, DNA replication, DNA repair, MYC targets, and PI3K/AKT/mTOR pathway, which was in agreement with the results of the KEGG and GO (Figure 4C). We also found the upregulation of cell response to hypoxia, which can lead to an increase in tumor invasiveness. The OCLR algorithm was subsequently applied to calculate mRNAsi (Malta et al., 2018). A higher mRNAsi score was found in the high-risk group, reflecting the loss of cell differentiation phenotype and acquisition of stem cell-like characteristics (Figure 4D). Based on single-cell sequencing data, we performed GSVA to analyze the pathway enrichment in HCC malignant cells of high- and low-risk samples (Figure 4E). A series of cancer-promoting pathways in the high-risk samples were upregulated, such as oxidative phosphorylation, MYC targets, and DNA repair. In contrast, low-risk samples showed increased activity of more cancer-inhibiting pathways, such as the P53 pathway, apoptosis process, and IL2-STAT5 signal pathway. The analysis results of bulk RNA-Seq and single-cell RNA-Seq revealed that a higher risk score predicted stronger proliferative ability and malignancy in HCC.

### 3.5 Genomic changes of cuproptosis and copper homeostasis related signature

**FIGURE 5:**
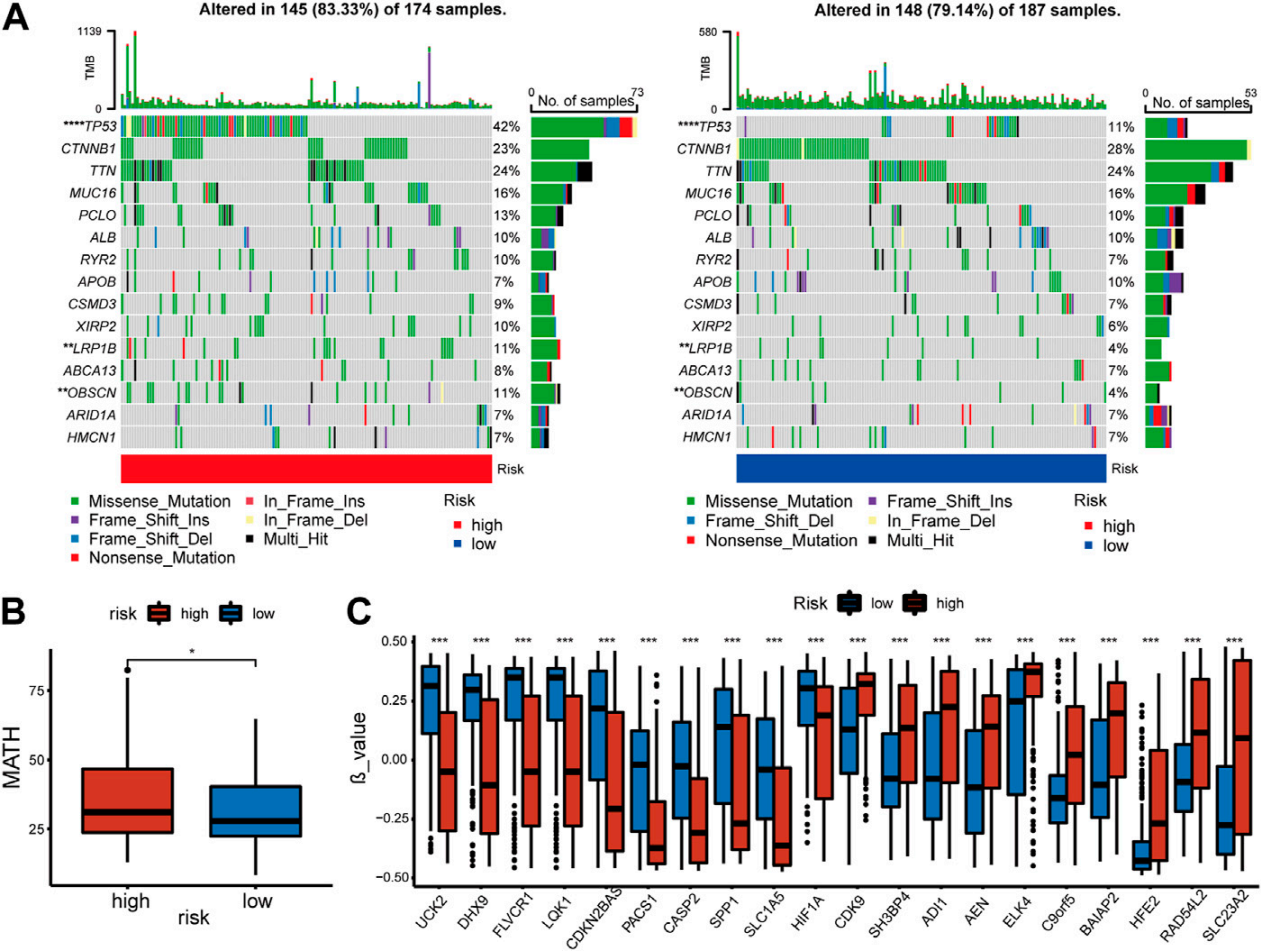
Features of mutation and methylation of high- and low-risk groups in the TCGA-cohort. **(A)** Diagrams of the 15 most substantially changed genes in the high-risk and low-risk subgroups. **(B)** The MATH scores of HCC patients from high-risk and low-risk subgroups. **(C)** The top 10 genes with the most significant positive or negative β value difference in high- and low-risk groups.

An investigation of the relationship between somatic mutations and the signature was conducted in high-risk and low-risk patients. The fifteen genes with the highest mutation rate were identified (Figure 5A). In spite of the fact that there was no significant difference in tumor mutation burden between groups with high- and low-risk scores (Supplementary Figure S1C), there were differences in tumor heterogeneity (Figure 5B) and mutation rates of several high-frequency mutant genes. High-risk individuals exhibited higher mutation rates of TP53 (*p* = 0), LRP1B (*p* = 0.008), and OBSCN (*p* = 0.008), all of which were identified as crucial tumor suppressors.

In addition, the methylation of genes was compared between the high and low-risk groups [Supplementary Table S1 (S3)]. β value was used to measure the methylation level of genes. The top 10 genes with the most significant positive or negative β value differences were displayed respectively (Figure 5C). A higher level of methylation was found in the high-risk group for the following genes: SH3BP4, ADI1, AEN, ELK4, C9orf5, BAIAP2, HFE2, RAD54L2, and SLC23A2. Methylation levels of the following genes were more significant in the low-risk group: UCK2, DHX9, FLVCR1, LQK1, CDKN2BAS, PACS1, CASP2, SPP1, SLCA5, HIF1a. The complete data was shown in [Supplementary Table S1 (S2)]. Finally, we compared copy number variations (CNV) in two groups, but no significant difference was found in the results (Supplementary Figure S1D).

### 3.6 Immune landscape analysis revealed immunosuppressive tendency of high-risk score sample

**FIGURE 6:**
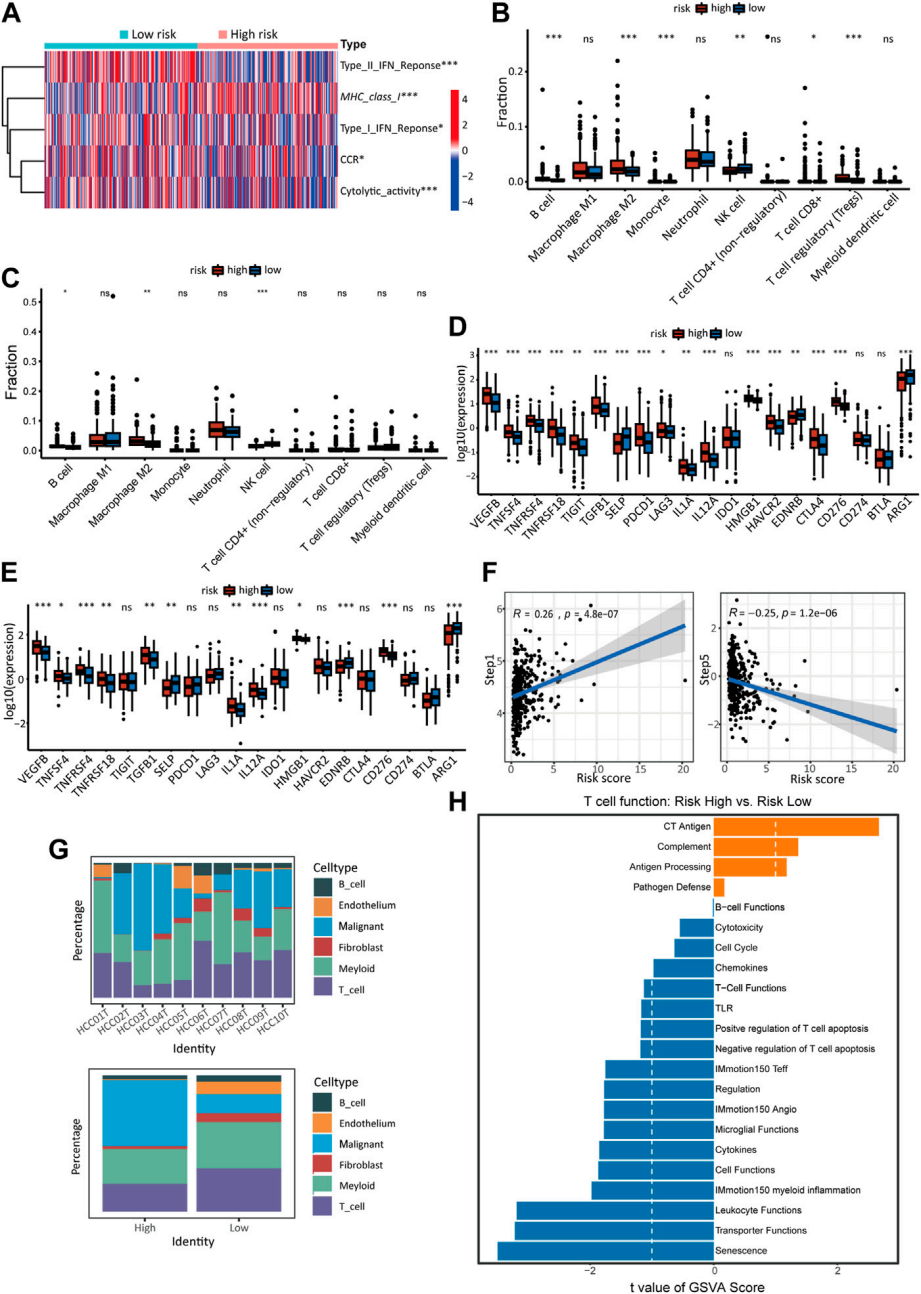
The immune landscape of high- and low-risk groups. **(A)** Heatmap of ssGSEA scores in the activity of immune-related processes in TCGA cohort. The quantiseq method calculates the proportion of 10 types of immune cells in low- and high-risk score groups of the TCGA cohort **(B)** and ICGC cohort **(C)**. The expression of immune checkpoints in the high-risk and low-risk groups of the TCGA cohort **(D)** and ICGC cohort **(E)**. **(F)** Correlation between cancer-immunity cycle scores and risk scores in the model in the TCGA cohort. **(G)** The proportion of malignant cells and immune-related cells in high-risk and low-risk samples of single-cell sequencing data. Detailed data was shown in supplementary Table S1 (S4). **(H)** GSVA analysis of T cell function in the single-cell cohort.

The TCGA cohort’s immune-related processes’ scores were calculated using ssGSEA (Figure 6A). The results showed decreased response to IFN-1 and IFN-2, decreased CCR activity, decreased cytolytic activity, and increased expression of MHC-1 in high-risk HCC patients. Using the quantiseq algorithm, the immune score of tumor tissue was quantified to further reveal the effect of different risk scores on the immune-related TME (Figure 6B). The high-risk group showed significant increases in B cells, M2 macrophages, monocytes, and T cells, but a decrease in NK cells. The immune infiltration in the external validation cohort (ICGC) was analyzed using the same method. The high-risk group showed significant increases in B cell and M2 macrophage, but a decrease in NK cells (Figure 6C), which was roughly in line with the TCGA cohort. Also, immune checkpoint molecules were examined that inhibit immune cells and allow tumors to escape immune recognition. A significant increase in the expression of most chosen immune checkpoint molecules (17/20) was observed in high-risk individuals (Figure 6D). The ICGC cohort also revealed significant differences in immune checkpoint expression in different risk groups (12/20) (Figure 6E), which confirms the TCGA cohort’s results. Analysis of the relationship between cancer immune cycle and risk score was carried out using TIP (http://biocc.hrbmu.edu.cn/TIP) (Figure 6F) (Xu et al., 2018). Risk scores and step 1 (antigen release from cancer cells) of the immune process were positively correlated, but step 5 (immune cell infiltration into tumors) was negatively correlated.

In the analysis of the sc-RNA data, We compared the contents of tumor cells and different types of immune cells in different groups. It was found that samples at high risk contained a higher proportion of malignant cells and a lower proportion of immune cells and other cells. (Figure 6G). T cells in TME are essential participants in tumor-related immune processes but are usually inhibited by various signals. T cells from high-risk and low-risk groups were compared using GSVA to investigate whether risk score impacts T cell function in TME (Figure 6H). The low-risk group showed significantly higher activity in T cell activation pathways than the high-risk group, such as cytotoxicity, chemicals, T cell functions, negative regulation of T cell apoptosis, IMmotion150 teff, cytokines, IMmotion 150 myoid inflammation, leucocyte function.

### 3.7 Ligand–receptors pairs analysis between immunocytes and HCC cells

**FIGURE 7:**
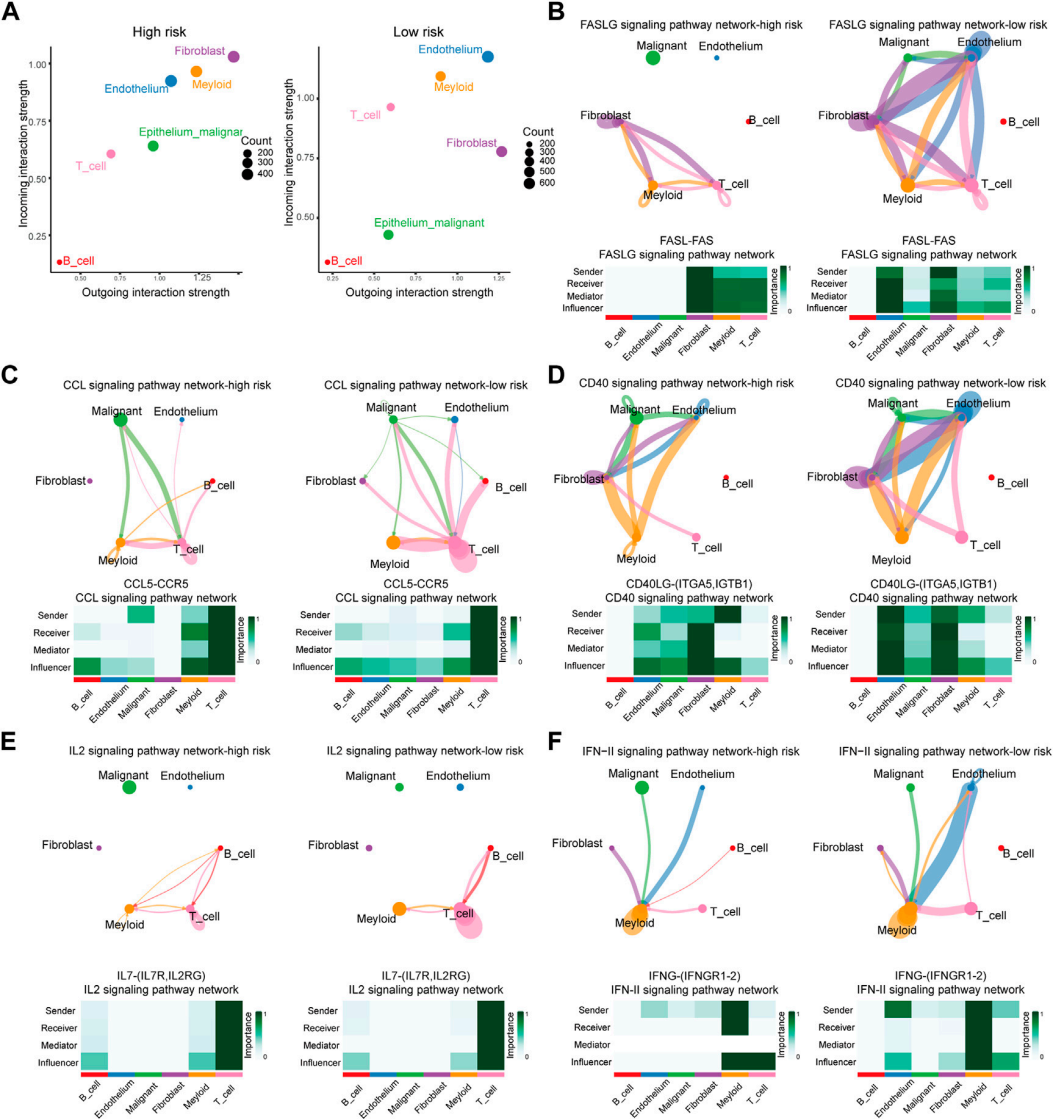
Differences of Ligand-Receptors in cell communication between high- and low-risk samples of single-cell RNA-Seq. **(A)** Dot graphs show how intensively each cell type communicates in high-risk and low-risk samples. **(B)** FASLG signaling network of high- and low-risk samples. **(C)** CCL signaling network of high- and low-risk samples. **(D)** CD40 signaling network of high- and low-risk samples. **(E)** IL2 signaling network of high- and low-risk samples. **(F)** IFN-II signaling network of high- and low-risk samples.

**FIGURE 8:**
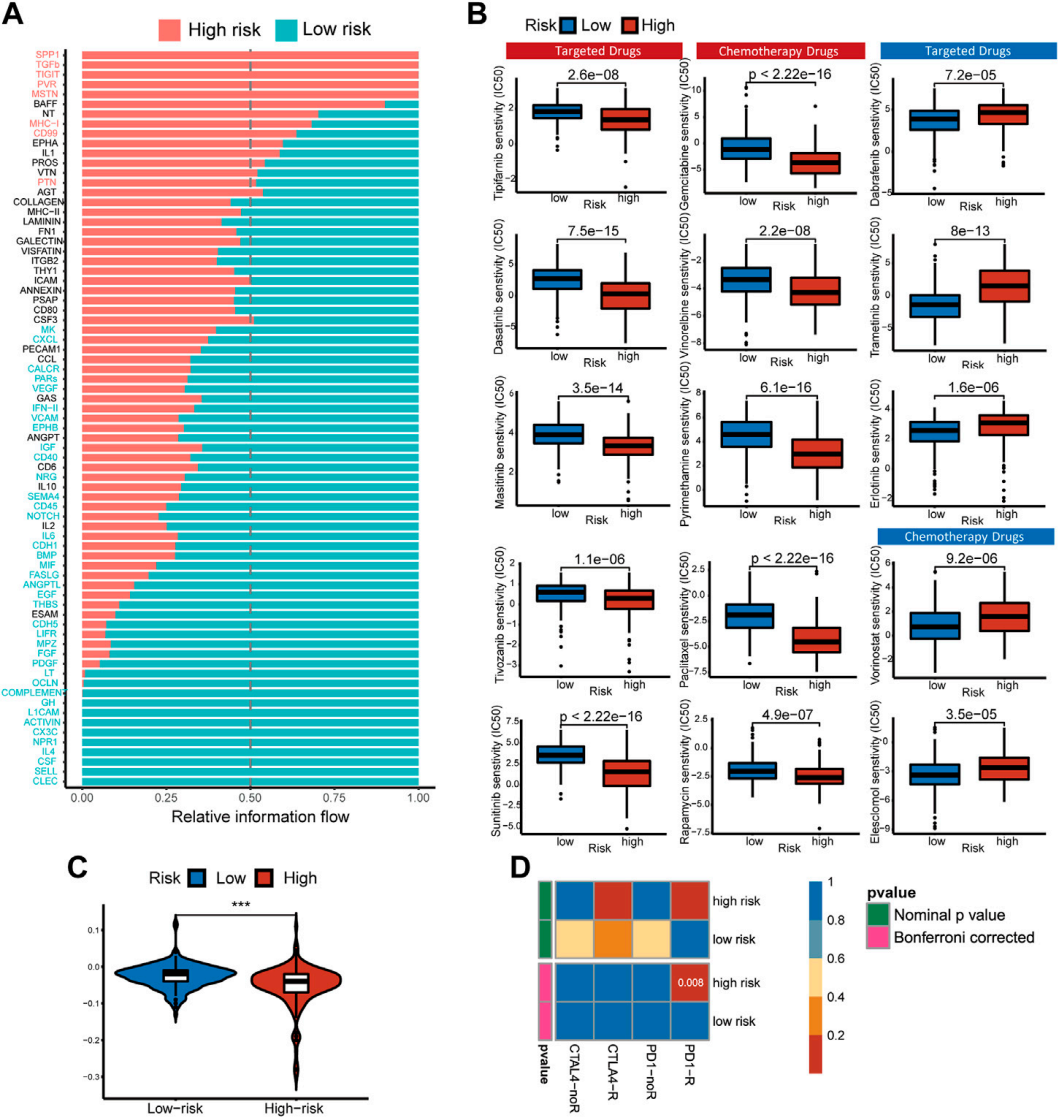
Pathways with different overall information between high- and low-risk single-cell sequencing samples. Predicting treatment response. **(A)** Signaling pathways with significant differences in the overall intercellular information between single-cell RNA-Seq samples at high- and low-risk. **(B)** The IC50s of chemotherapeutic agents and targeted drugs related to the 5-gene signature. **(C)** The prediction results show the distribution of immune response scores in the high- or low-risk groups of the TCGA cohort. **(D)** Differences in sensitivity of subgroups based on risk score to immunotherapy. Submap classing analysis manifested that the high-risk score group could be more sensitive to the programmed cell death protein 1 (PD-1) inhibitor (Bonferroni-corrected *p* = 0.008).

The analysis above revealed that the infiltration rate of immune cells was different between high- and low-risk scores. As a result, we conducted a communication analysis between malignant cells and other immune-related cells based on the single-cell sequencing data to find the pathways and corresponding targets (Figure 7A). In the high-risk samples, fibroblasts dominated signal input and output. In comparison, the signal input of the low-risk samples was dominated by endothelial cells. In the communication between tumor cells and other cells, Signal intensity and communication process were significantly different between high- and low-risk groups for the following pathways: FASLG (FASL-FAS) signal pathway (Figure 7B), CCL (CCL5-CCR5) signal pathway (Figure 7C), CD40 (CD40L-(ITFA5, IGTB1)) signal pathway (Figure 7D), IL2 (IL7R-IL7RG) signal pathway (Figure 7E), and IFN-II (IFNG-(IFNGR1-2)) signal pathway (Figure 7F). The high-risk group has different degrees of signal intensity reduction in these pathways, the activation of which could assist in the anti-tumor process. The total information flow between high-risk and low-risk groups also differed significantly across other signaling pathways (Figure 8A). To a certain extent, this explains the decrease in immune cell infiltration and the tendency of immunosuppression in the HCC TME of high-risk samples.

### 3.8 Responses prediction of chemotherapeutic and immune therapy

Non-operative treatment of HCC faces the challenge of drug resistance, and copper has been proven to alter tumor cell drug resistance. To evaluate risk characteristics’ role in clinical treatment, we compared high- and low-risk patients’ sensitivity to chemotherapeutics and target therapy. In total, 156 differential drugs and molecular compounds were screened out, with 127 drugs being more sensitive to the high-risk group and 29 drugs being more sensitive to the low-risk group [Supplementary Table S1 (S5)]. The IC50 value estimated for low-risk cancer patients is higher than that found in low-risk cancer patients for the following clinically common targeted therapy drugs: Tipifarnib, Tivozanib, Masitinib, Dasatinib, Sunitinib, and chemotherapy drugs: Gemcitabine, Vinorelbine, Rapamycin, Paclitaxel, Pyrimethamine (Figure 8B).

Based on TIDE algorithm, immune checkpoint inhibitor responses in different patient groups were predicted. The TIDE score was higher in low-risk patients than in high-risk patients, which means the efficacy of immune checkpoint blocking therapy (ICB) was worse, and the survival time was shorter after ICB treatment (Figure 8C). Based on the IPS of HCC patients, the response to immunotherapy targeted specifically at CTLA-4 and PD-1 in high-risk and low-risk HCC patients was examined using a subclass mapping approach. The high-risk patients responded well to anti-PD-1 therapy, while the low-risk patients had no reaction to either anti-PD-1 or anti-CTLA4 therapy. (Figure 8D). We found that the treatment options mentioned above were more likely to benefit high-risk patients, regardless of whether they were traditional chemotherapy or targeted therapy.

### 3.9 Verification of biological function and expression level of model-constructed genes

**FIGURE 9:**
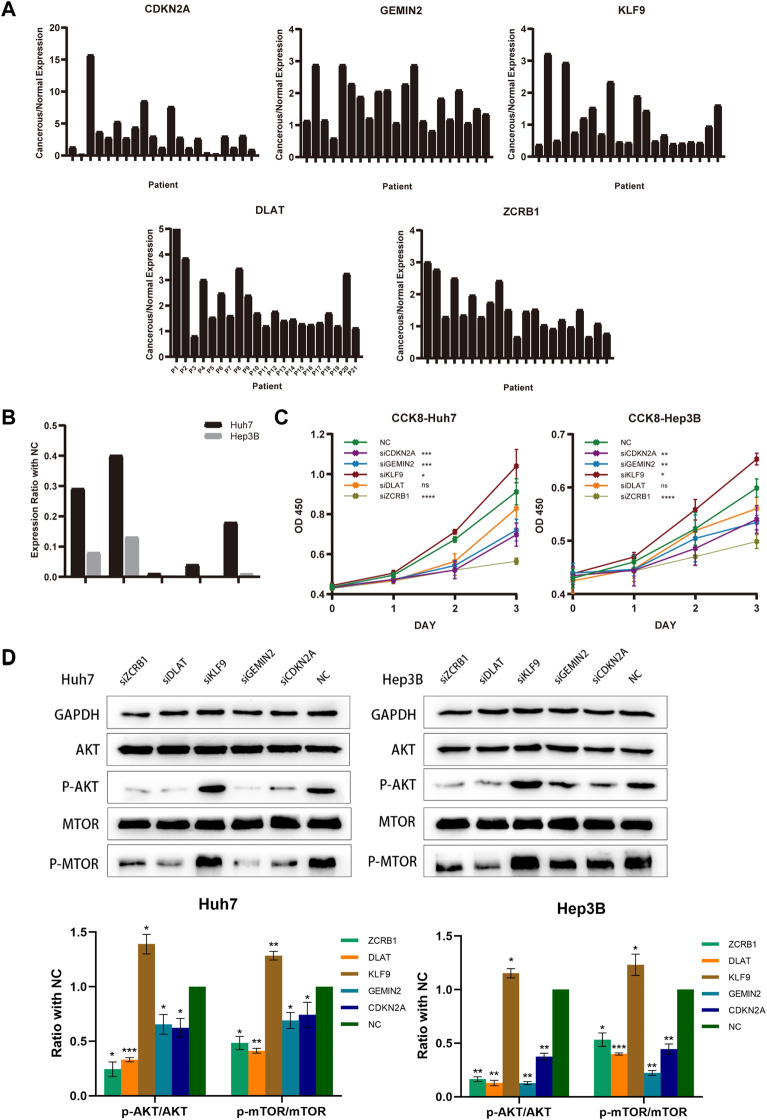
Model-constructed genes expression in HCC tissues and effects on HCC cell biological signature. **(A)** qRT-PCR analysis of model-constructed gene expression in paired tumors and adjacent normal tissues from 21 HCC patients. **(B)** The efficiency of transfection was determined by qRT-PCR. **(C)** CCK-8 was used to determine growth curves for transfected Huh7 and Hep3B cellsB cells. **(D)** AKT, p-AKT, mTOR, and p-mTOR expression in different groups were detected with western blot.

Twenty-one HCC patients were tested using qRT-PCR on paired tumors and normal adjacent tissues. CDKN2A, GEMIN2, DLAT, and ZCRB1 were expressed at higher levels in tumors than in normal tissues, while the expression of KLF9 in tumor tissue was lower (Figure 9A). For the subsequent study, we selected Hep3B and Huh7 cell lines transfected with sh-RNAs for knockdown experiments of five model-constructed genes. The plasmid transfection efficiency of all five genes for both cell lines was greater than 50% (Figure 9B). The proliferation and viability of cells were assessed by the CCK-8 assay. Knockdown of CDKN2A, GEMIN2, and ZCRB1 prominently impaired cell growth of both Huh7 and Hep3B, while the knockdown of KLF9 improved the cell growth of both cell lines (Figure 9C).

According to previous findings (Figure 4C), different risk groups differed significantly in proliferative capacity and activity of the PI3K/AKT/mTOR signal pathway, which is widely implicated in mitochondrial metabolism and tumor drug resistance. There is evidence that phosphorylation of AKT and mTOR affects copper-induced disease progression in a variety of diseases, including cancer. The results showed that the knockdown of CDKN2A, GEMIN2, DLAT, and ZCRB1 prominently impaired the phosphorylation of both AKT and mTOR in Huh7 and Hep3B, while the knockdown of KLF9 improved the phosphorylation (Figure 9D).

### 3.10 *In Vitro* effects of ZCRB1 on biological behavior of liver cancer cells

**FIGURE 10:**
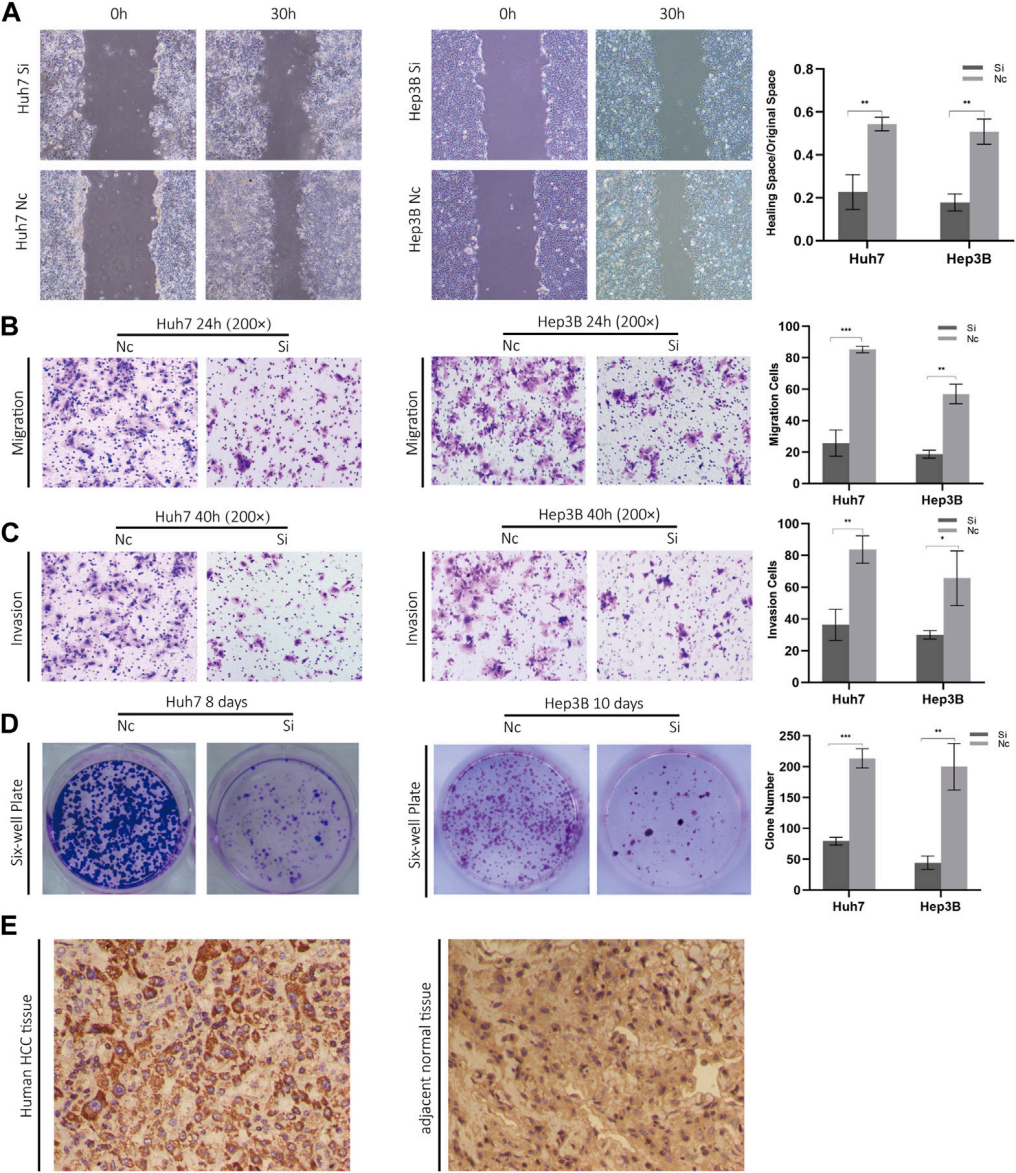
**(A)** Scratch experiments were used to determine migration and wound healing. **(B)** Transwell migration assays. **(C)** Transwell invasion assays. **(D)** Clonogenic assays**. (E)** Phenotypic experiments on ZCRB1 *in vitro* and immunohistochemistry results in clinical samples.

Considering the knockout of ZCRB1 had the strongest tumor-inhibiting effect in the proliferation experiment, specifically, we selected ZCRB1 as the target to determine its effect on proliferation, invasion, migration, and colony formation. In the scratch assay experiment, scratches in the knock-down group healed slower than in the control group (Figure 10A). In addition, transwell invasion and migration experiments confirmed that ZCRB1 downregulation significantly reduced tumor cell invasion and migration (Figures 10B, C). ZCRB1 also inhibited the growth of Huh7 and Hep3B colonies after knockdown, suggesting that ZCRB1 boosts colony formation (Figure 10D). Besides, we performed immunohistochemical analyses of human HCC tissues and adjacent normal tissue using ZCRB1 antibody. The expression of ZCRB1 in HCC tissue was significantly stronger than in adjacent normal tissue. The expression of ZCRB1 in HCC tissue was significantly stronger than in adjacent normal tissue Figure 10E. Through the Human Protein Atlas (HPA) database, we also supplied the immunohistochemical images of the remaining model-constructed molecules in the HPA database, which indicated that there was a higher expression of GEMIN2, CDKN2A, and DLAT in HCC than in normal tissues (Supplementary Figure S1E), but the immunohistochemical data of KLF9 were not obtained. As a result, *in vitro* experiments suggest that ZCRB1 expression is closely related to malignant behavior in tumor cells, and it could become a new therapeutic target for copper homeostasis and cuproptosis.

## 4 Discussion

Compared with other trace elements in the human body, copper has unique redox activity, making it an essential catalytic cofactor (Kim et al., 2008). Copper homeostasis disorder can lead to intracellular copper overload, leading to cellular protein toxic stress, which is the reason for acute cell death in cuproptosis (Tsvetkov et al., 2022). During cell proliferation, copper participates in the signal cascade (Tsang et al., 2020), promotes proliferation and diffusion, and participates in tumor microenvironment changes (Soncin et al., 1997). The critical role of copper uptake, distribution, and effluent ligand/pump expression in cancer has been confirmed (Itoh et al., 2008; Blockhuys et al., 2017; Blockhuys et al., 2020). Because of copper’s role in cancer development and the crucial position of the liver in the process of copper storage and metabolism, we searched for the related genes of copper homeostasis and cuproptosis through correlation analysis and then constructed a copper homeostasis and cuproptosis associated gene signature by Lasso regression and multivariate COX analysis. HCC patients’ prognoses could be well predicted with the model, and clinical characteristics were combined with risk scores to construct a nomogram model for facilitating clinical research and application. Several studies have examined the relationship between cuproptosis and patient prognosis in HCC (Ding et al., 2022; Peng et al., 2022; Xie et al., 2022; Zhang et al., 2022). For example, Peng et al. developed a prognostic model based on cuproptosis-related genes (Peng et al., 2022). Xie et al. built a cuproptosis-related immune checkpoint gene signature to identify the prognosis of HCC patients (Xie et al., 2022). Ding et al. also built a cuproptosis-related prognosis model and discussed it in different cuproptosis subtypes (Ding et al., 2022). Before the concept of cuproptosis was proposed, copper and copper homeostasis had been revealed to be related to many diseases, including cancers. Cuproptosis is mainly involved in participants of the TCA cycle within mitochondrial metabolism while maintaining intracellular copper homeostasis requires an intracellular multi-structure system. No research has been conducted based on copper homeostasis in hepatocellular carcinoma or other diseases. In fact, the integrity role of copper homeostasis and cuproptosis in disease has been recognized. A recent study published on signal transport and target therapy comprehensively elaborated on broad application prospects of copper homeostasis and cuproptosis and proposed that reliable biomarkers are scarce as of now (Chen et al., 2022). Besides, the clinical utility of specific models is crucial. Decision Curve Analysis (DCA) is a method to evaluate and compare multiple clinical prediction models in clinical utility, which was proposed by Dr. Andrew Vickers. This method allows us to compare our study with other cuproptosis-related models. At the time of 1, 3, and 5 years, our model exhibits better application value than the models based solely on cuproptosis (Supplementary Figure S1F). Additionally, our study was the first to explore in detail the function of tumor cells and T cells as well as the intercellular communication among different risk groups based on single-cell sequencing data, which clarifies the impact of copper-related physiological processes on different components in tumor microenvironments and provides a new perspective for follow-up readers’ studies.

Previous studies have shown that copper in the TME can directly or indirectly activate metalloenzyme function and oxidative stress (Ma et al., 1999). Without regular intracellular disposal, excessive oxidative stress will induce tumor cell transformation and uncontrolled proliferation (Hsu et al., 1994). In the functional analysis of the TCGA cohort and single-cell RNA-Seq samples, high-risk patients and malignant cell clustering showed higher proliferative capacity and viability under hypoxia. The risk score in malignant cell clustering is significantly higher than in others. Furthermore, patients with high-risk scores had higher mRNAsi scores, reflecting an acquired stem cell-like phenotype and loss of cell differentiation. The high-risk group had an increased mutation rate for TP53, LRP1B, and OBSCN. A previous study showed that OBSCN is an effective tumor suppressor in various cancers (Guardia et al., 2021). It is also known that TP53 mutation frequency is higher in cancers with increased malignancy. LRP1B is a tumor suppressor, but LRP1B-mutated cancers have improved outcomes with ICIs, the underlying mechanism of which has not yet been clarified (Brown et al., 2021). In addition, many genes with different methylation levels are associated with proliferation. This explains in one way why patients with high-risk scores had poorer clinical outcomes, indicating that the genes related to copper homeostasis and cuproptosis affect the tumor proliferation and malignancy in HCC and may even be involved in forming cancer stem cells.

Cuproptosis is mainly involved in participants of the TCA cycle within mitochondrial metabolism, while mitochondrial metabolism and glycolysis are highly related to the phosphorylation of AKT (Stiles, 2009). Early studies have confirmed that tumorigenesis can be reduced by inhibiting the copper transporter 1-copper axis *via* AKT signaling (Chen et al., 2021; Guo et al., 2021). And before cuproptosis was revealed, some studies had previously attempted to change cancer cells’ tolerance to specific drugs by blocking the activity of AKT (Banerjee et al., 2016; Wu et al., 2018). By knocking out the model-constructed genes, we revealed that the model-constructed genes were strongly correlated with AKT and mTOR phosphorylation levels. Despite the function of some crucial molecules has been proved, there are still unsolved mysteries. In a recent study, bioinformatics and experimental verification were combined to prove the effect of DLAT on AKT phosphorylation in HCC (Zhou et al., 2022). According to another study, CDKN2A-mediated AKT phosphorylation influences cervical cancer malignancy (Luan et al., 2021), but there are no relevant studies in HCC. KLF9 is downregulated in HCC, which could stabilize p53 and induce apoptosis (Sun et al., 2014), while it remains unknown whether it affects the activity of AKT-related pathways in HCC. A particular interest of ours is ZCRB1, which is an RNA-binding protein. As a tumor suppressor gene, ZCRB1 phosphorylates JMJD5 to regulate aerobic glycolysis in GBM through the cyclic RNA HEATR5B (Song et al., 2022). There are few studies on the role of ZCRB1 in cancer. According to our results, however, knocking out ZCRB1 significantly inhibits the malignant phenotype of HCC, as well as inhibiting the phosphorylation of AKT and mTOR. Considering the heterogeneity of copper-related metabolic processes in different tissues, ZCRB1 may combine different circRNAs and complete the phosphorylation of AKT/mTOR through different signal axes. And this process may be caused by an imbalance in copper homeostasis or cuproptosis.

Copper participates in human immunity, which promotes leukocyte differentiation, maturation, and proliferation and maintains the phagocytosis of neutrophils (Djoko et al., 2015). The role of copper in antitumor immunity has been demonstrated in recent studies. In the immune regulation of cancer, disulfiram as a copper carrier can make cancer cells carry excess copper and maintain the stability of PD-L1 in HCC (Zhou et al., 2019). In the immune-activation mouse model of neuroblastoma (GBM), copper chelation therapy with TEPA can reduce the PD-L1 expression of GBM, improve the anti-GBM immune response mediated *via* NK cells, and inhibit the immune checkpoint (Voli et al., 2020). These studies advise that reducing the concentration of copper in the tumor can stimulate the anti-cancer immune response and promote new immune cell clones in tumors. We found that the immune cells infiltrating the tumor tissues of high-risk patients were significantly reduced. The proportion of NK cells decreased, while immunosuppressive cells (Macrophage M2, Tregs) increased significantly. Changes in pathways related to immune cells and chemokines indicate that it is believed that copper accumulation in TME reduces the ability of immune cells to infiltrate and weakens the body’s immune response to malignant cells as a result.

In the TME, T cells play a significant role in anti-tumor immunity. Besides being a target for immune checkpoint therapy, it can also promote tumor immune escape, so understanding its characteristics is crucial (Oh et al., 2021). Based on single-cell sequencing data, GSVA was performed on T cells of samples from different risk groups, and many pathways related to T cell activity were suppressed, which suggests T cell activity may be regulated by genes involved in copper homeostasis and cuproptosis. Furthermore, positive correlations were found between several immune checkpoints and risk scores. As shown above, immunosuppression tends to be more common in high-risk patients, and the score of the signature reflects that tendency.

Our analysis of single-cell sequencing data revealed that the difference in cell communication between different cells could be the mechanism behind TME changes associated with the signature. An array of signaling pathways and corresponding receptor-ligand pairs were identified. Fas is found in virtually all cells, while the FasL gene is predominantly expressed in activated T cells. Inducing apoptosis and cell death is the primary function of Fas/FasL. T cells and NK cells trigger tumor cell apoptosis through FasL, a tumor suppressor gene (Villa-Morales and Fernández-Piqueras, 2012). CCL5’s role in tumors has been controversial. Some studies suggest that its production induces immunosuppression (Chang et al., 2012), while others suggest it promotes tumor immunity (Harlin et al., 2009; Liu et al., 2015). Tumor necrosis factor (TNF) receptors include the CD40 receptor. CD40 activates dendritic cells, which then activate CD8 + T cells Vonderheide, 2020. Monoclonal CD40 has shown efficacy in tumor therapy (Cancer Discov, 2017). In addition to proliferating effector T cells, IL-2 regulates the growth of Treg cells. IL-2-based anticancer treatments are becoming increasingly popular (Mullard, 2021). Anti-CTLA-4 resistance is affected by the expression of IFNG1 (Cancer Discov, 2017), whereas anti-PD-1 resistance is affected by the expression of IFNG2 (Williams et al., 2020). The signal intensity of the above pathways and their receptor-ligand pairs decreased to varying degrees in high-risk samples. Taking into account the change in T cell activity, the above ligand-receptor pairs may be required for genes related to copper homeostasis and cuproptosis to participate in communicating intercellularly, which may begin with the activation of immune-helper cells, such as dendritic cells, followed by the activation of effector T cells such as CD8^+^ and NK cells. Consequently, this will lead to a change in immune-related TME and ICBs sensitivity.

As a traditional therapy for HCC, chemotherapy can’t wholly remove tumor cells because of inherent or acquired drug resistance (Siddik, 2003). Applying copper-based complexes and copper-chelating agents is sufficient to bypass cisplatin resistance in different types of cancer (Mo et al., 2018; Rochford et al., 2020; Vančo et al., 2021). Similar methods were used in clinical trials of breast and prostate cancers (Henry et al., 2006; Pass et al., 2008; Chan et al., 2020). In several studies, chemotherapy resistance was associated with the downregulation of copper transporters and the upregulation of pumps and chaperones for copper efflux. (Katano et al., 2002; Safaei and Howell, 2005; Yu et al., 2020). The relationship between copper metabolism and chemotherapy resistance is disease-specific. For example, the clinical correlation between copper transporter 1(CTR1) expression and the efficacy of platinum chemotherapeutic drugs were contradictory in different studies (Ishida et al., 2010; Lee et al., 2011; Akerfeldt et al., 2017). Therefore, applying the risk model requires a prediction of the chemotherapeutic drug’s sensitivity. In our research, 127 drugs and compounds were expected to be sensitive to high-risk HCC patients. Additionally, patients at high risk responded better to immunotherapy targeting PD-1, which provides a reference for further research and clinical application. Considering the copper dependence on cancer progression and the low cytotoxicity of copper-chelating drugs (Hsu et al., 1994), it has the potential to use genes related to copper homeostasis and cuproptosis as immunotherapy targets. Due to the limitation of understanding the metabolic process of copper and the related mechanisms of copper homeostasis in different tumor drug resistance, no copper complexes have been used in anti-tumor therapy. More copper-related targets and pathways in cells must be found to develop more stable drug ligands. Our research provides a new application direction for traditional chemotherapeutic and targeted drugs.

## 5 Conclusion

A novel scoring model related to copper homeostasis and cuproptosis was developed in this study. High-risk scores predicted poor prognosis, high tumor malignancy, and tumor immunosuppression in HCC patients. Novel receptor-ligand pairs were proposed as targets for the changes in immune function and TME based on the intercellular communication status. Targeted and chemotherapeutic drugs with potential effects were predicted. Meanwhile, model-constructed genes were validated in terms of their clinical and functional significance, but further study is needed to understand the mechanism in more detail. As a result of our research, we are able to evaluate the malignant degree, TME changes, and cross-talk between malignant cells and immunocytes in patients with HCC, which can provide suggestions for treatment.

## Data Availability

The original contributions presented in the study are included in the article/Supplementary Material, further inquiries can be directed to the corresponding authors.

## Supplementary material

SUPPLEMENTARY FIGURE S1
**(A)** The cell markers used to determine cell types. **(B)** Quality control of single-cell sequencing data. **(C)** Comparison of tumor burden between high- and low-risk groups in TCGA cohort. **(D)** The six genes with the highest CNV frequency in the high- and low-risk groups of the TCGA cohort. **(E)** Immunohistochemical image of model-constructed genes from human protein atlas. GEMIN2 CA/NC sample number 46570; CDKN2A CA/NC sample number CAB000445; DLAT CA/NC sample number CAB003782. **(F)** DCA (Decision Curve Analysis) of three different prognostic models. The X-axis was determined by threshold probability, while net benefits were shown on the y-axis. Based on the TCGA-LIHC cohort, the clinical usefulness of the treatment was higher when the net benefit was higher under the same probability. Model 1: the model in this study. Model 2: The signature was constructed by Peng et al, which was based on cuproptosis-related genes (38). Model 3: the signature was developed by Zhang et al, which was based on the cuproptosis core gene FDX1 and its related genes (39). Model 4: the signature was developed by Xie, which was based on cuproptosis-related immune checkpoint genes (40). Model 5: the signature was developed by Ding, which was based on cuproptosis-related genes (41).

SUPPLEMENTARY TABLE S1
**S1–S16** **(S1)** Candidate genes confirmed as genes related to copper homeostasis and cuproptosis. **(S2)** The risk score of the single-cell sequencing samples. **(S3)** Differentially expressed genes (DEGs) between groups with high or low-risk scores in the TCGA cohort. **(S4)** The proportion of malignant cells and immune-related cells in high-risk and low-risk samples of single-cell RNA-Seq. **(S5)** The results of drug response prediction. (**S6, S7**) KEGG and GO enrichment analysis results with a P-value of 0.05 and a |FC| > 2. (**S8, S9**) The results of GSEA of proliferation-related function and immune process. (**S10**) The mRNAsi scores of patients in the TCGA cohort which was calculated with the OCLR algorithm. (**S11**) TCGA cohort immune checkpoint expression. **(S12, S13)** The TIDE scores of the TCGA cohort and the result of subclass mapping analysis. **(S14, S15)** The results of GSVA of malignant cells or T cells in the single-cell RNA-Seq. **(S16)** The result of intercellular communication analysis.

SUPPLEMENTARY TABLE S2 Raw data of CCK8 and RT-qPCR.

SUPPLEMENTARY TABLE S3 Primer sequence used in the experiment.

SUPPLEMENTARY DATA SHEET S1 Western blot image and strip quantization.

SUPPLEMENTARY DATA SHEET S2 Scratch assay image and analysis.

SUPPLEMENTARY DATA SHEET S3 Image and analysis for Migration, invasion, and clone formation.

SUPPLEMENTARY DATA SHEET S4 Image for IHC.

## Glossary

AIC: Akaike information criterion
AUC: Area under curve
CCL: C-C Motif Chemokine Ligand
CD40: CD40 molecule
CDKN2A: Cyclin dependent kinase inhibitor 2A
CTLA-4: Cytotoxic T lymphocyte-associated antigen-4
DLAT: Dihydrolipoamide S-Acetyltransferase
FASLG: Fas ligand
GDSC: The genomics of drug sensitivity in cancer database
GEMIN2: Gem nuclear organelle associated protein 2
GO: Gene ontology
GSVA: Gene set variation analysis
HCC: Hepatocellular carcinoma
ICB: Immune checkpoint blockade
IFN: Interferon
KEGG: Kyoto encyclopedia of genes and genomes
KLF9: Kruppel like factor 9
LASSO: Least absolute shrinkage and selection operator
LRP1B: LDL receptor related protein 1B
MATH: Mutant-allele tumor heterogeneity
OBSCN: Obscurin, cytoskeletal calmodulin and titin-Interacting RhoGEF
OCLR: One-class logistic regression
OS: Overall survival
PD-1: Programmed cell death protein 1
PFS: Progression-free survival
ROC curve: Receiver operating characteristic curve
ssGSEA: Single sample gene set enrichment analysis
TIDE: Tumor immune dysfunction and exclusion
TME: Tumor microenvironment
TNM: T-Tumor, N-regional lymph mode, M-Metastasis
TP53: Tumor protein P53
UMAP: Uniform Manifold Approximation and Projection
ZCRB1: Zinc finger CCHC-Type and RNA binding motif containing 1.
