# Supplementary figures and images for: A novel prognostic scoring model based on copper homeostasis and cuproptosis which indicates changes in tumor microenvironment and affects treatment response
Source: Front Pharmacol. 2023 Feb 24;14:1101749. doi: 10.3389/fphar.2023.1101749 (PMC9998499; doi:10.3389/fphar.2023.1101749)

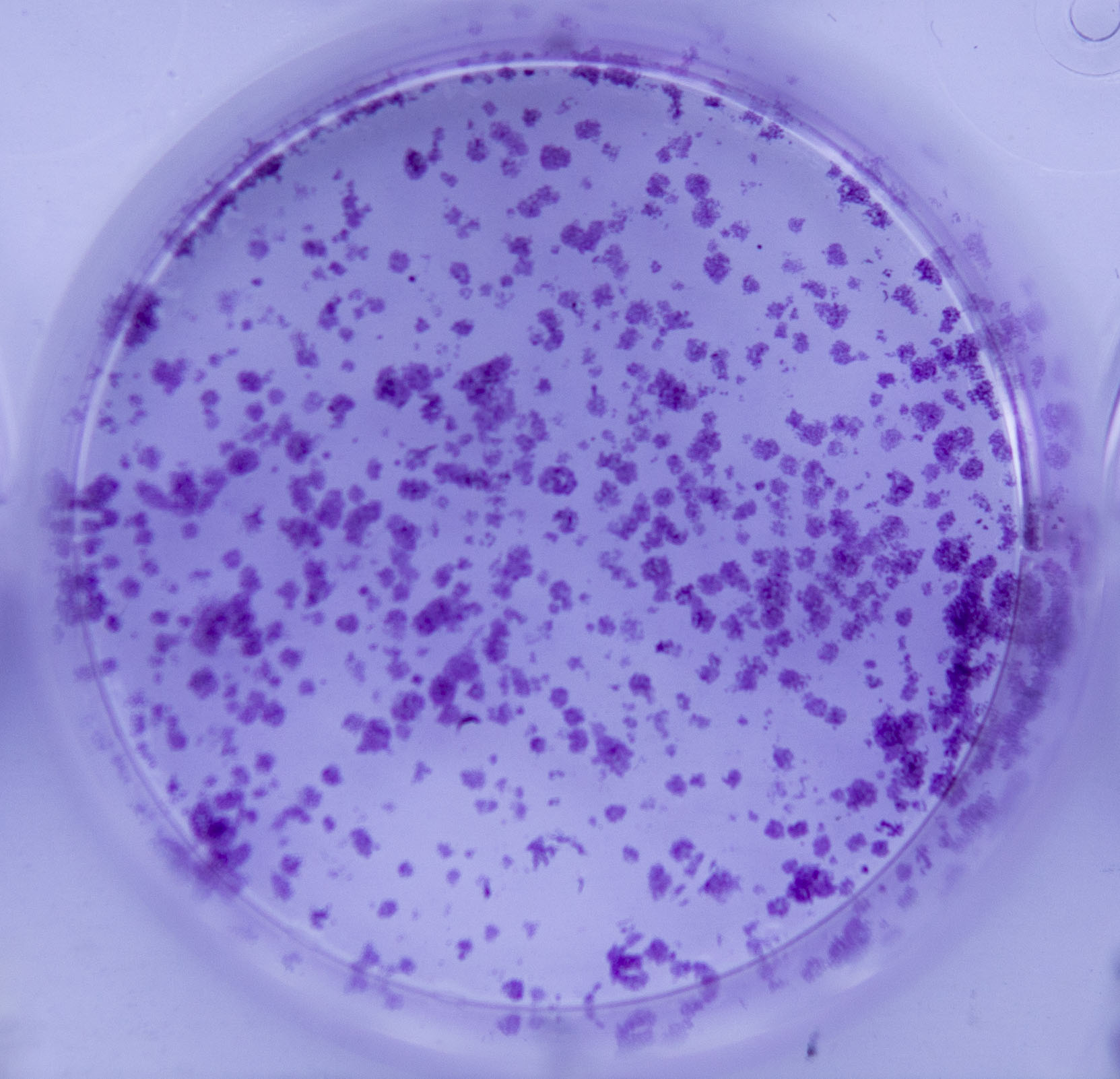

Supplement: Supplementary file 2 [file DataSheet3.ZIP › Hep3B NC.jpg]

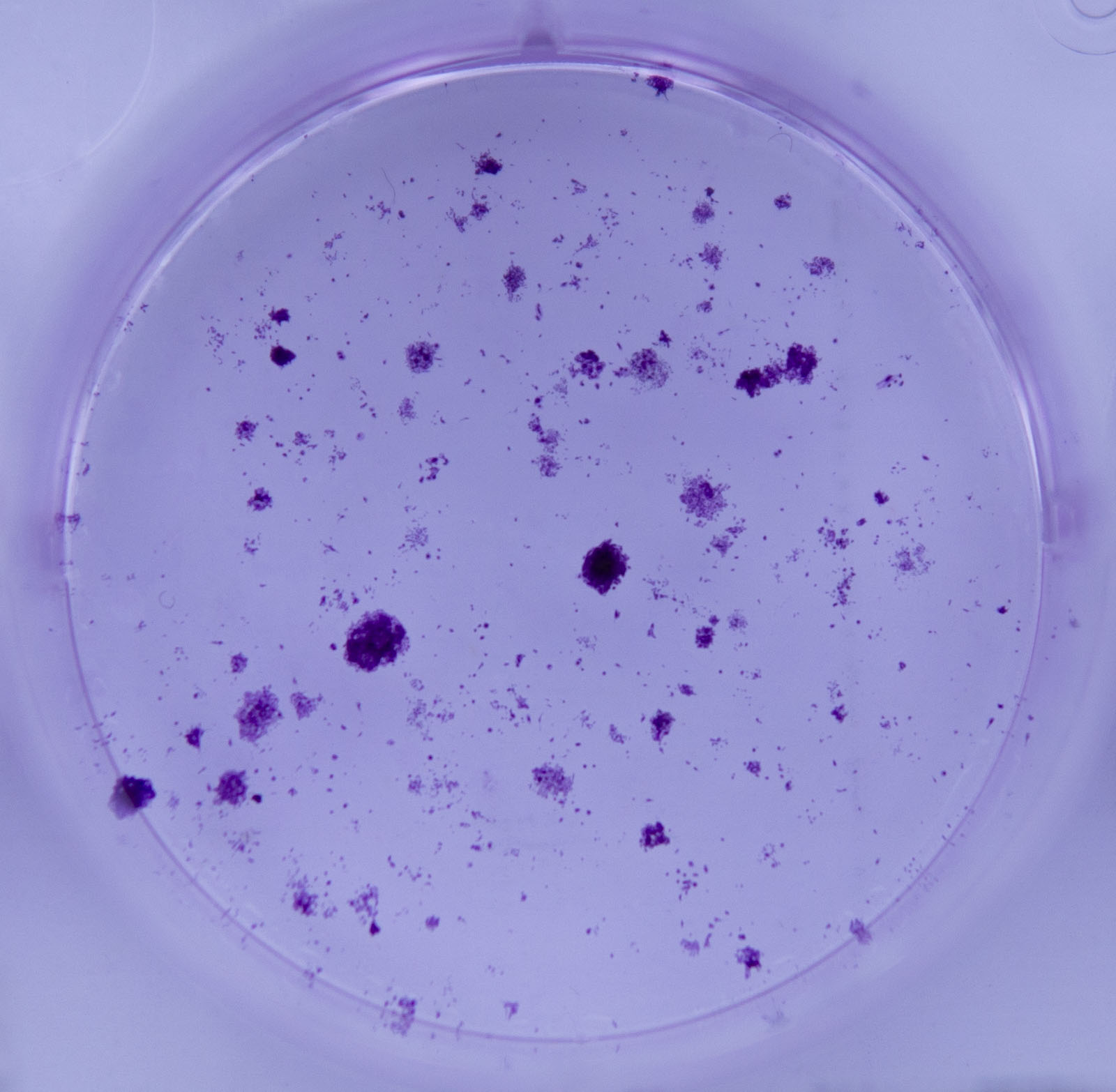

Supplement: Supplementary file 2 [file DataSheet3.ZIP › Hep3B Si.jpg]

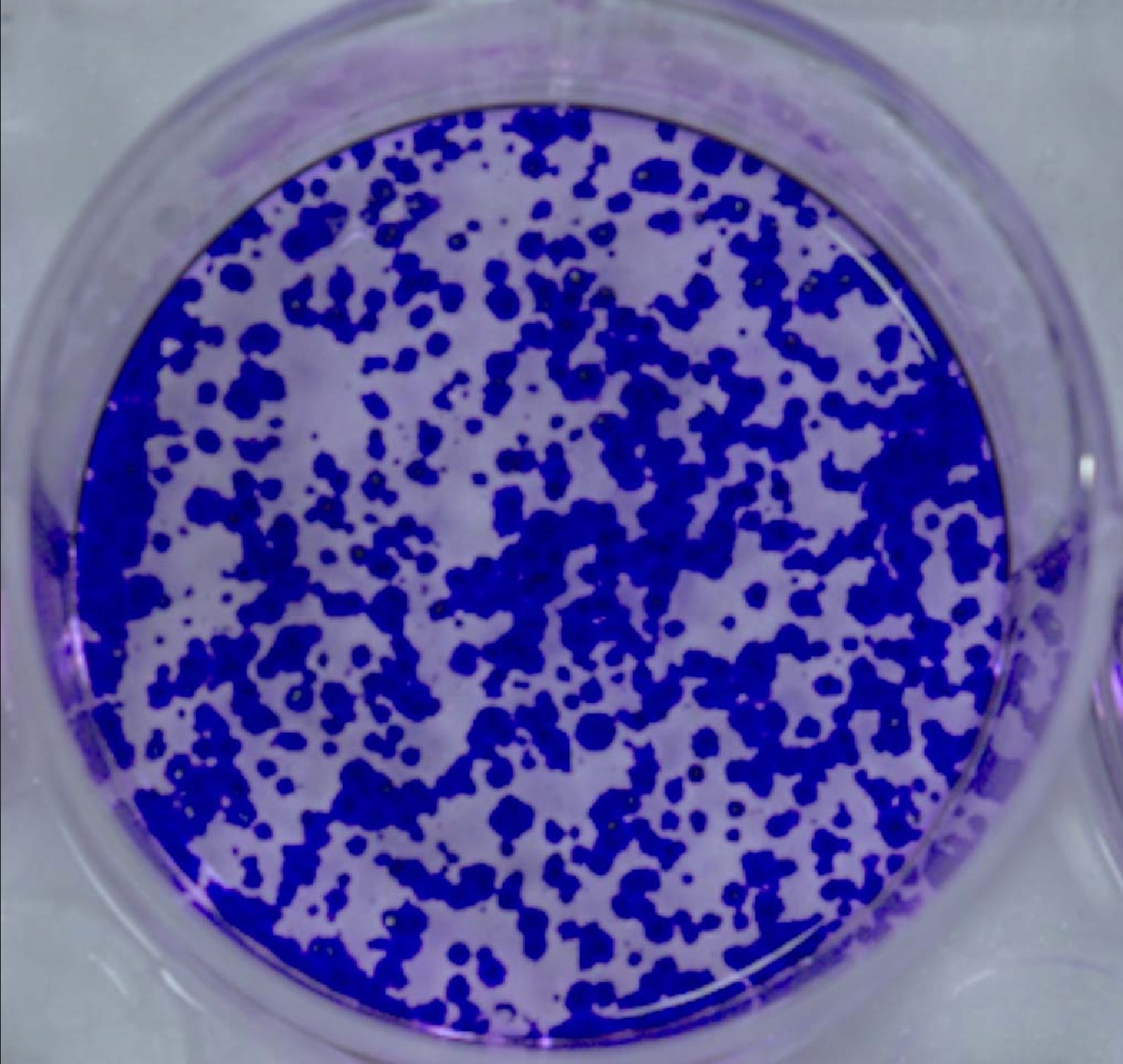

Supplement: Supplementary file 2 [file DataSheet3.ZIP › Huh7 NC.jpg]

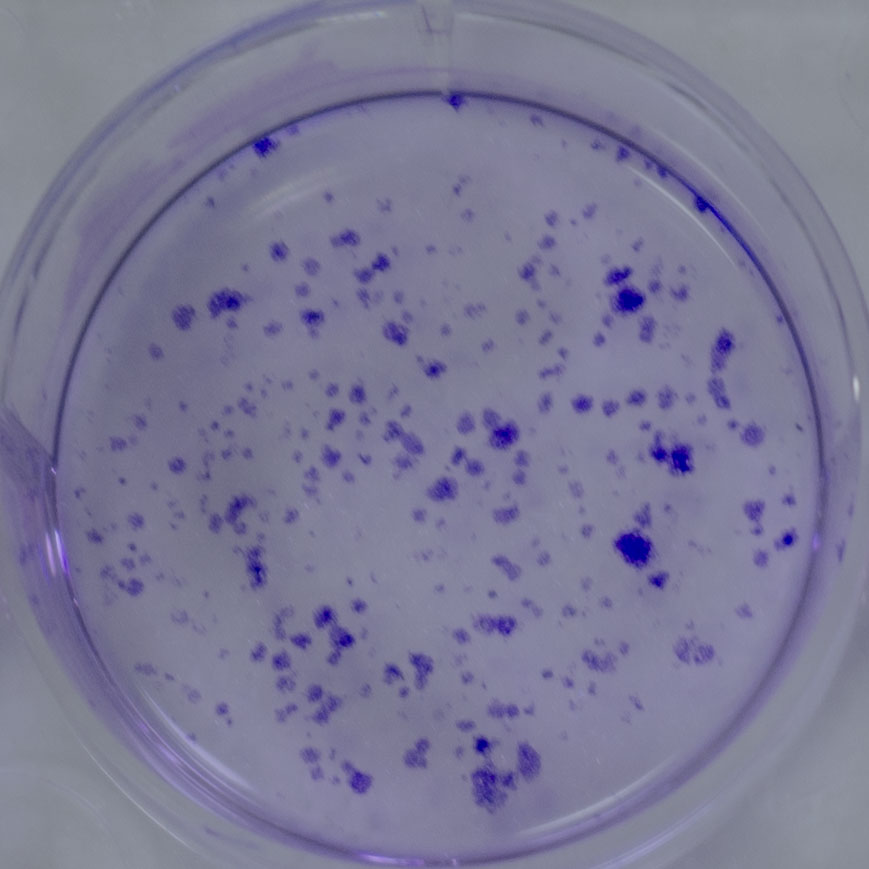

Supplement: Supplementary file 2 [file DataSheet3.ZIP › Huh7 Si.jpg]

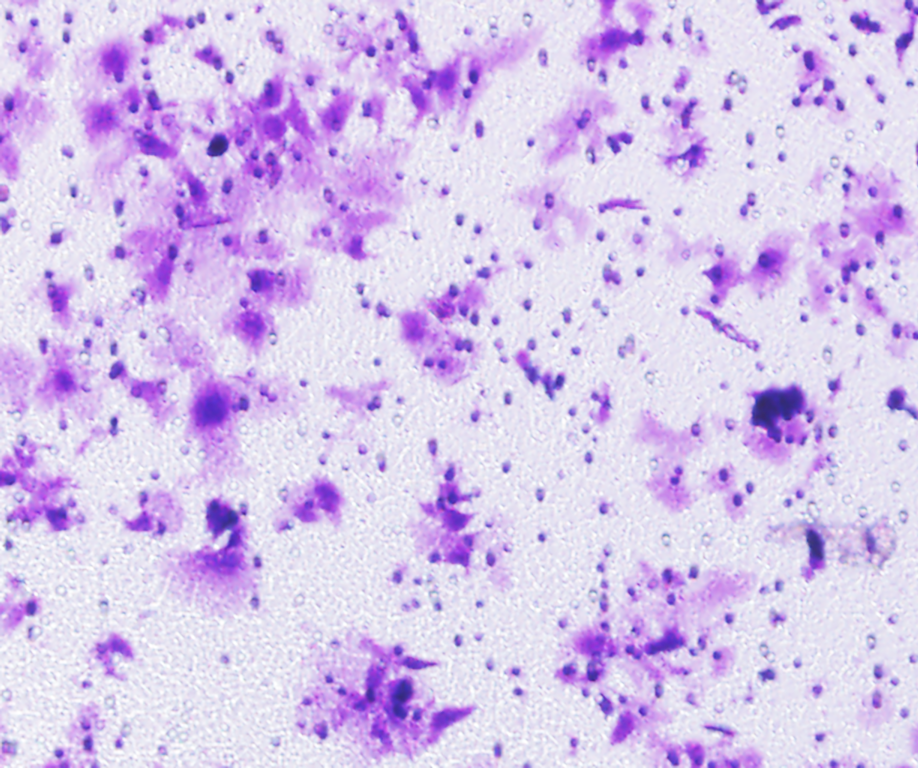

Supplement: Supplementary file 2 [file DataSheet3.ZIP › Invasion Hep3B NC.png]

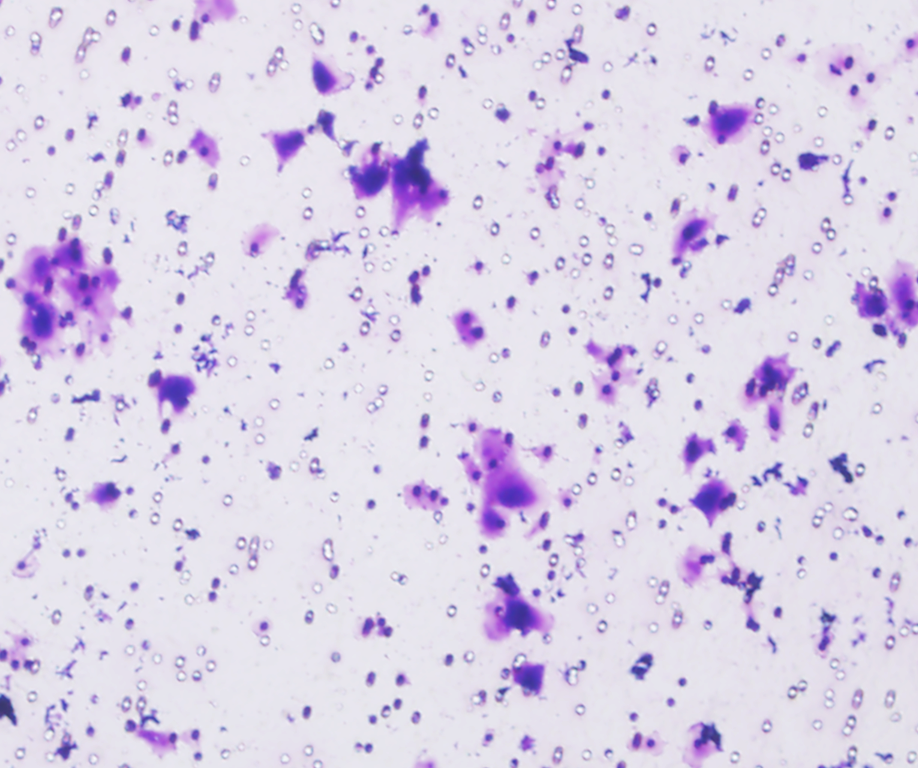

Supplement: Supplementary file 2 [file DataSheet3.ZIP › Invasion Hep3B Si.png]

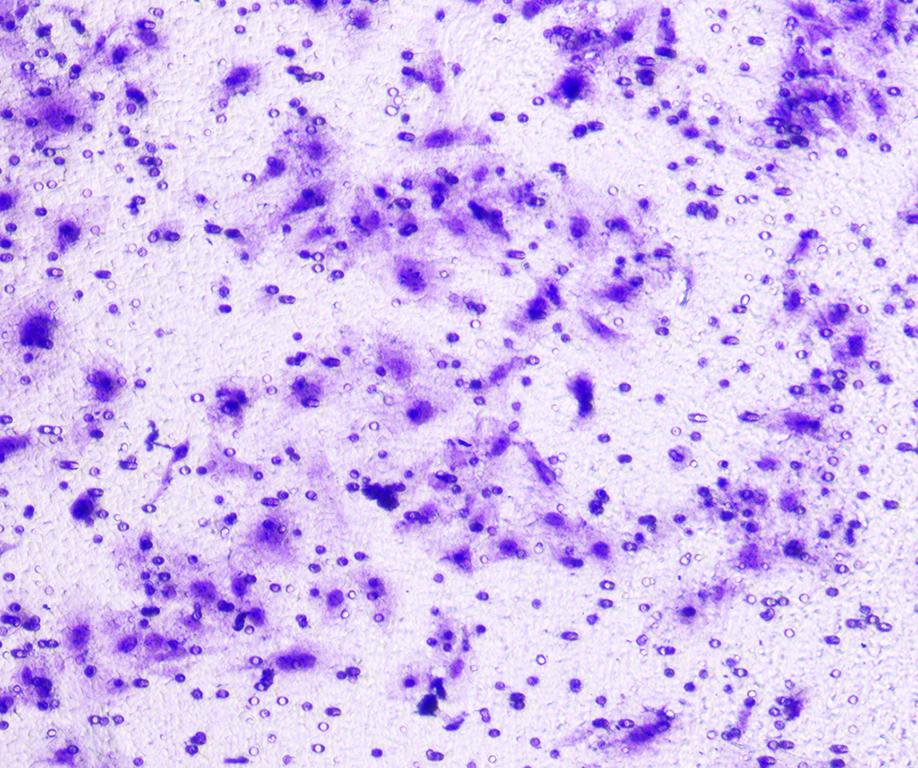

Supplement: Supplementary file 2 [file DataSheet3.ZIP › Invasion Huh7 Nc .png]

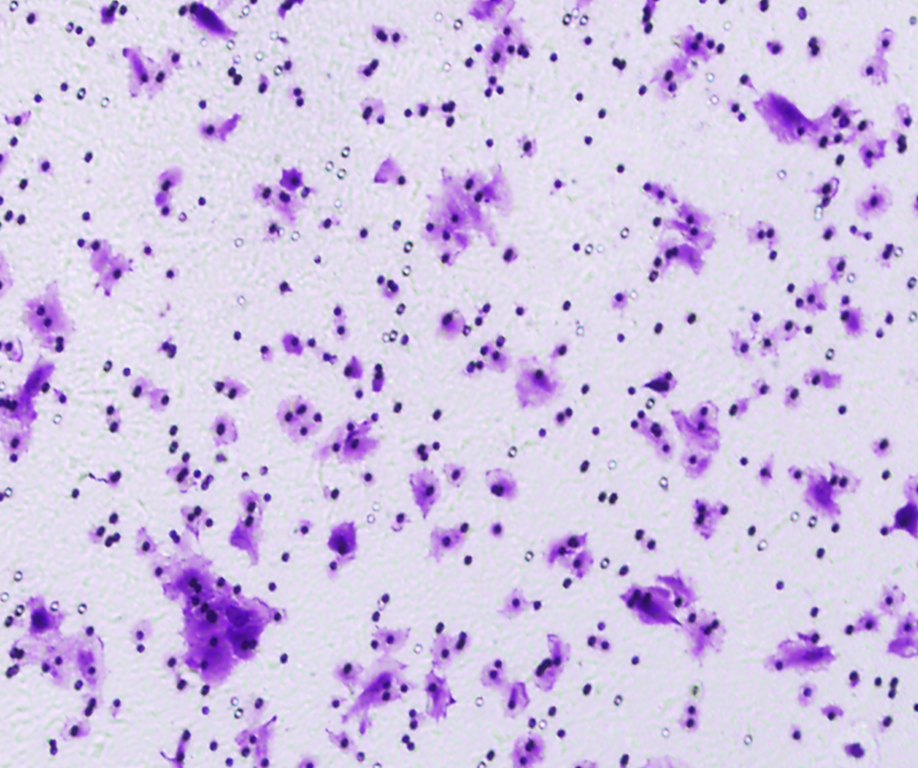

Supplement: Supplementary file 2 [file DataSheet3.ZIP › Invasion Huh7 Si.png]

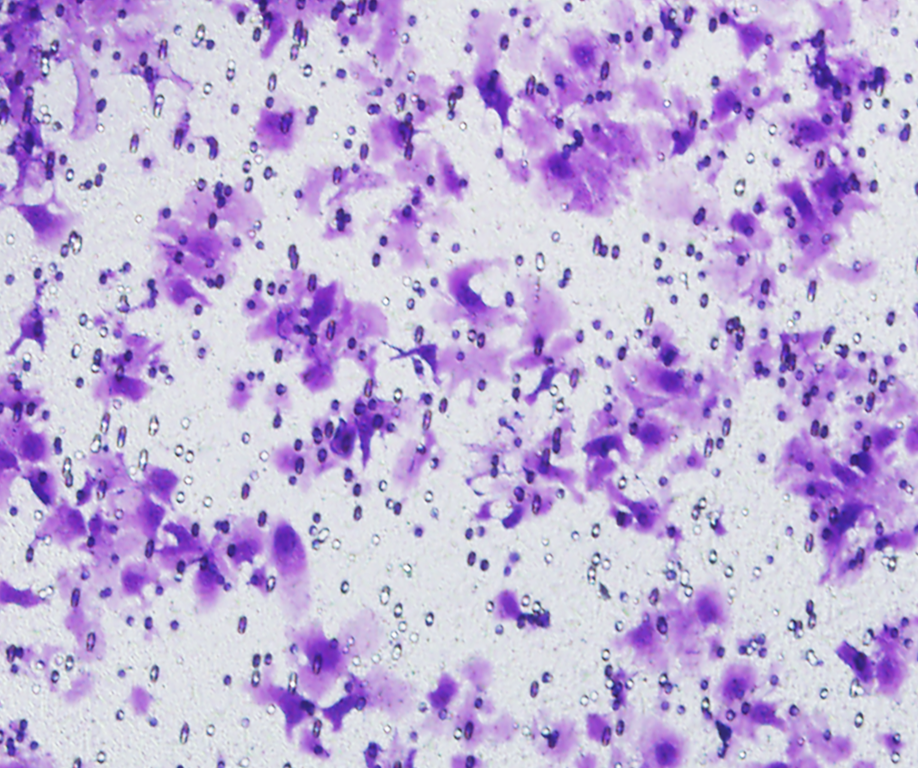

Supplement: Supplementary file 2 [file DataSheet3.ZIP › Migration Hep3B NC.png]

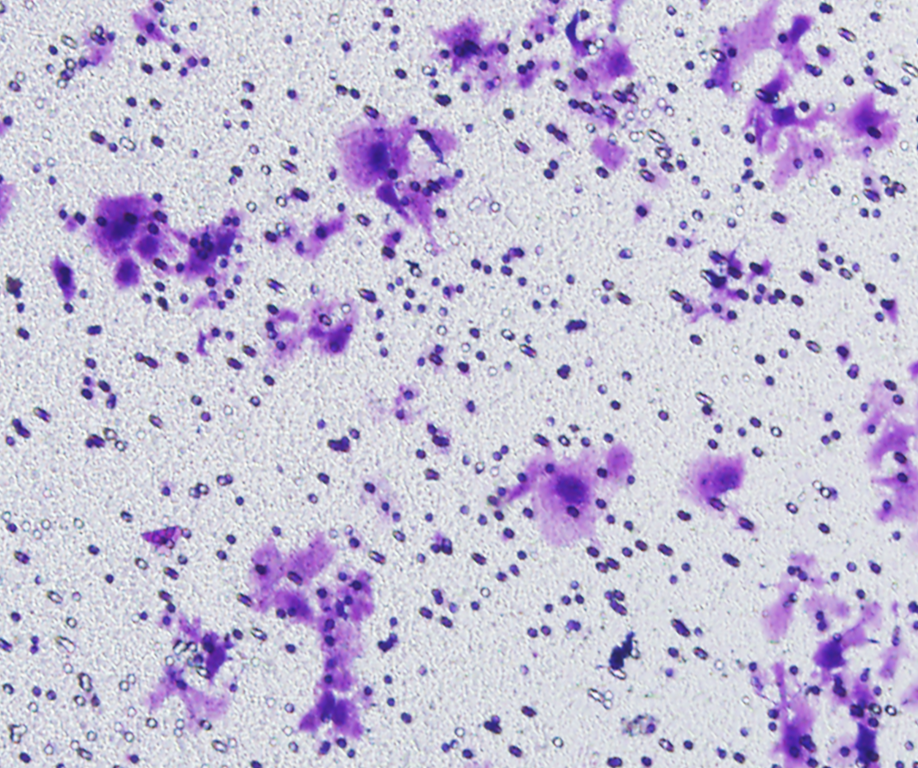

Supplement: Supplementary file 2 [file DataSheet3.ZIP › Migration Hep3B Si.png]

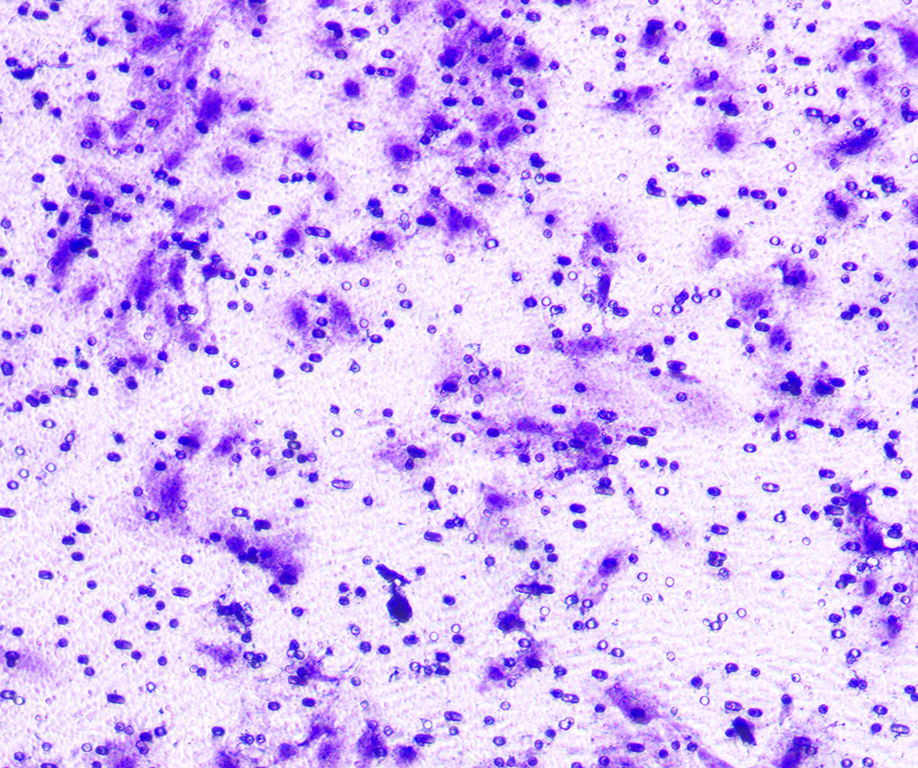

Supplement: Supplementary file 2 [file DataSheet3.ZIP › Migration Huh7 NC .png]

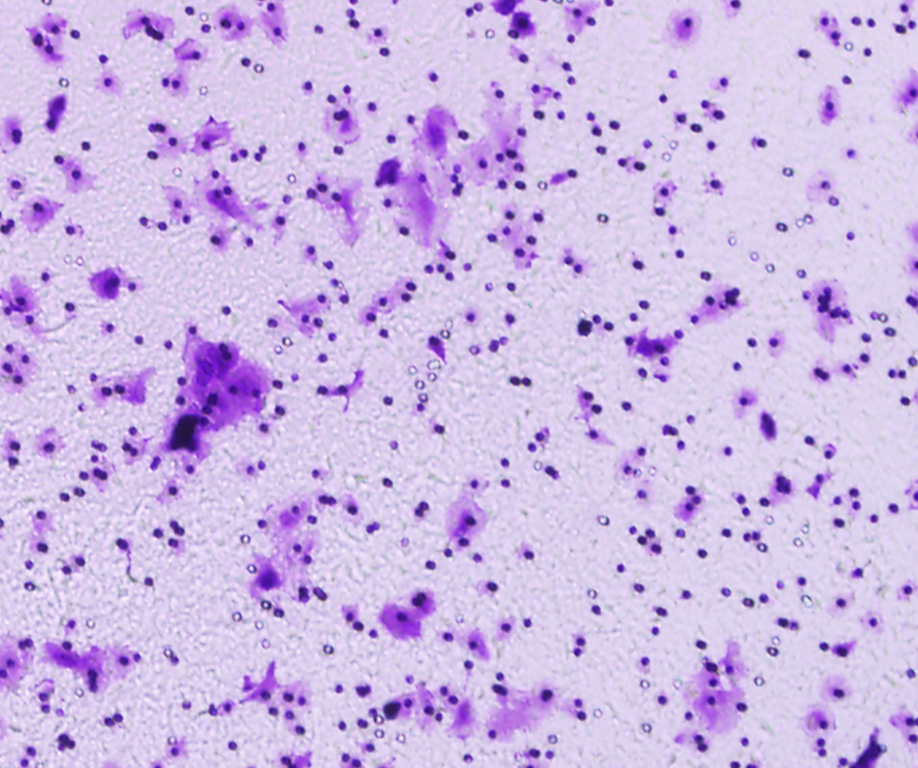

Supplement: Supplementary file 2 [file DataSheet3.ZIP › Migration Huh7 Si .png]

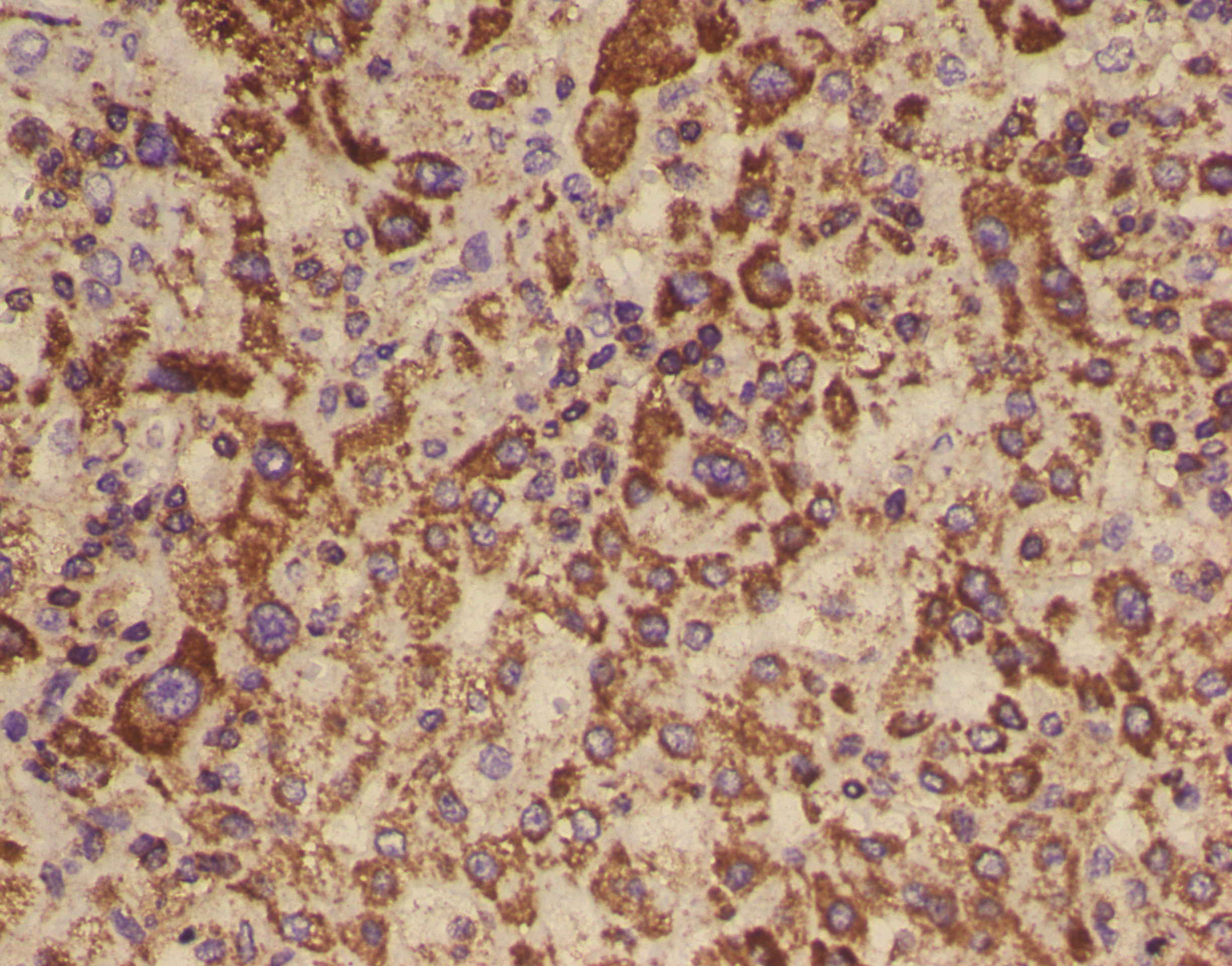

Supplement: Supplementary file 4 [file DataSheet4.ZIP › IHC CA 200.tif]

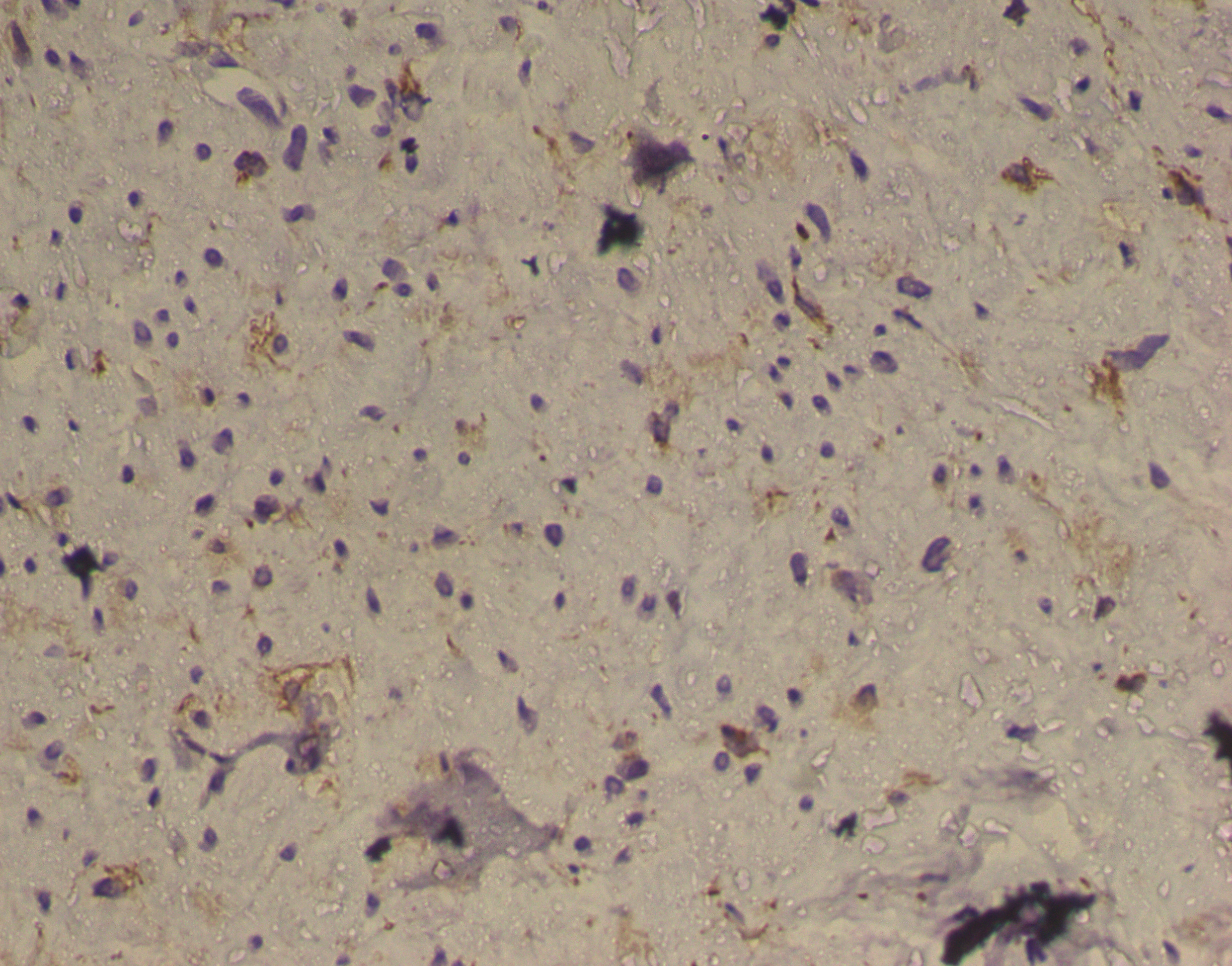

Supplement: Supplementary file 4 [file DataSheet4.ZIP › IHC NC 200.tif]

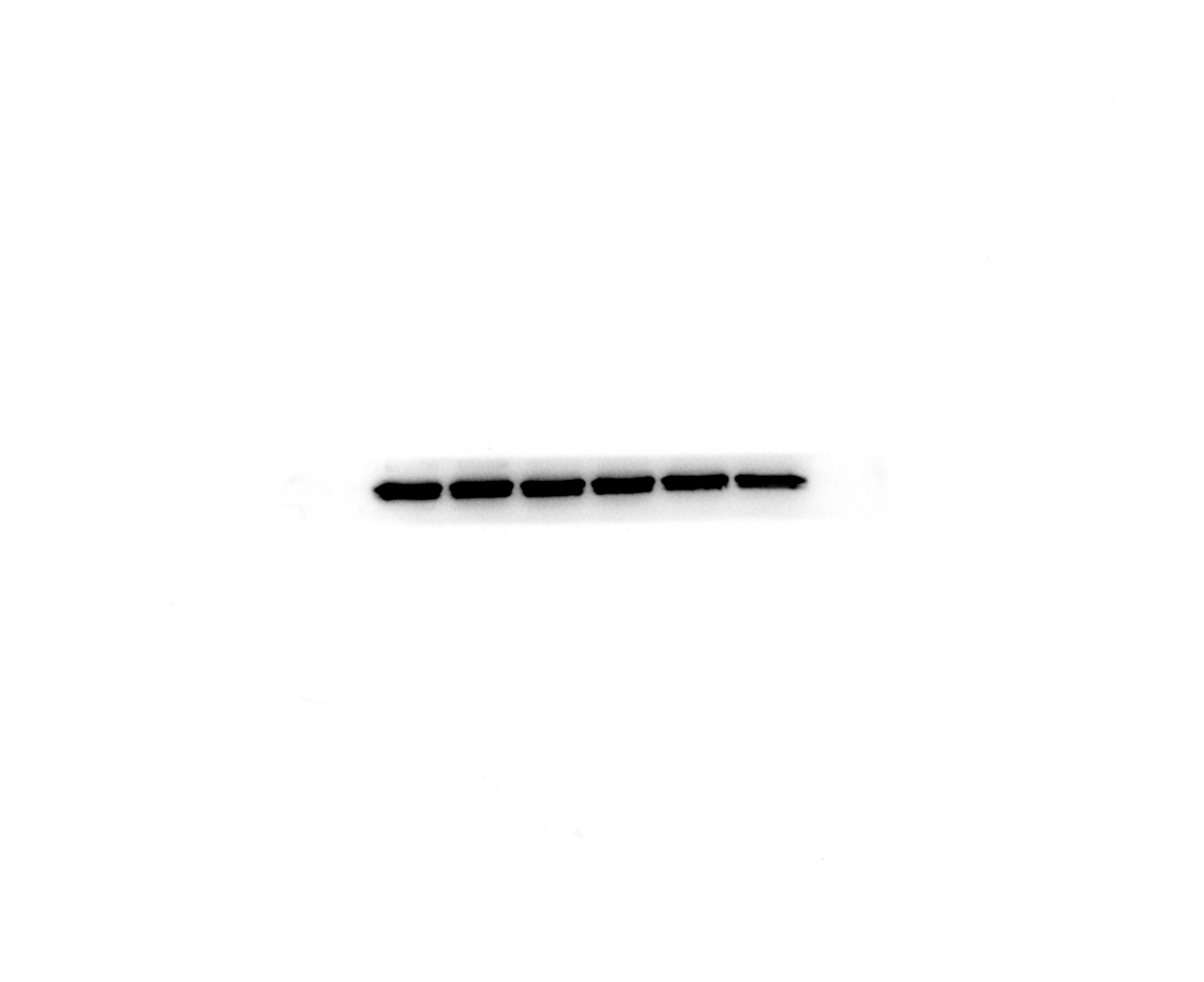

Supplement: Supplementary file 5 [file DataSheet1.zip › Hep3B/AKT.Tif]

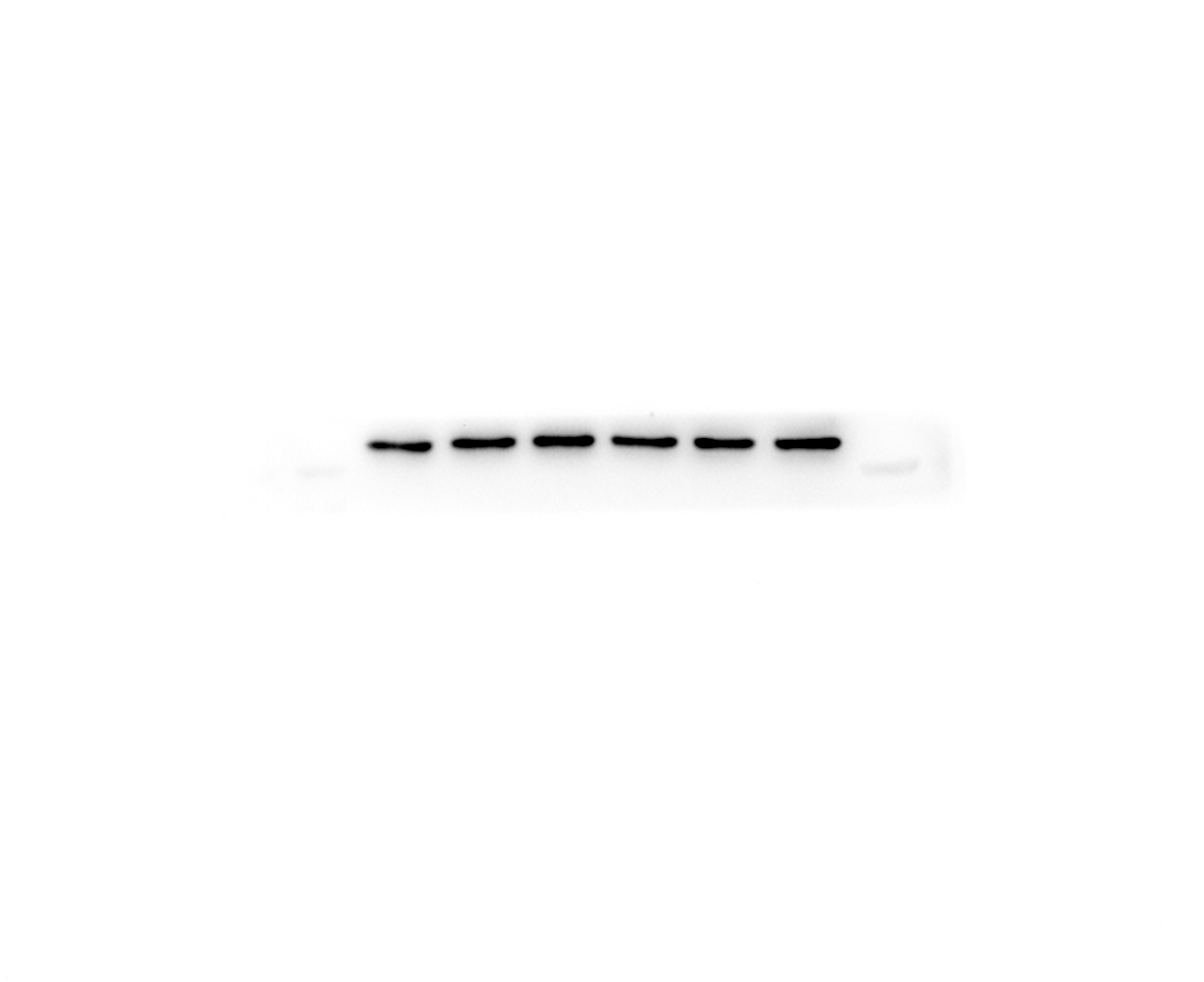

Supplement: Supplementary file 5 [file DataSheet1.zip › Hep3B/GAPDH.Tif]

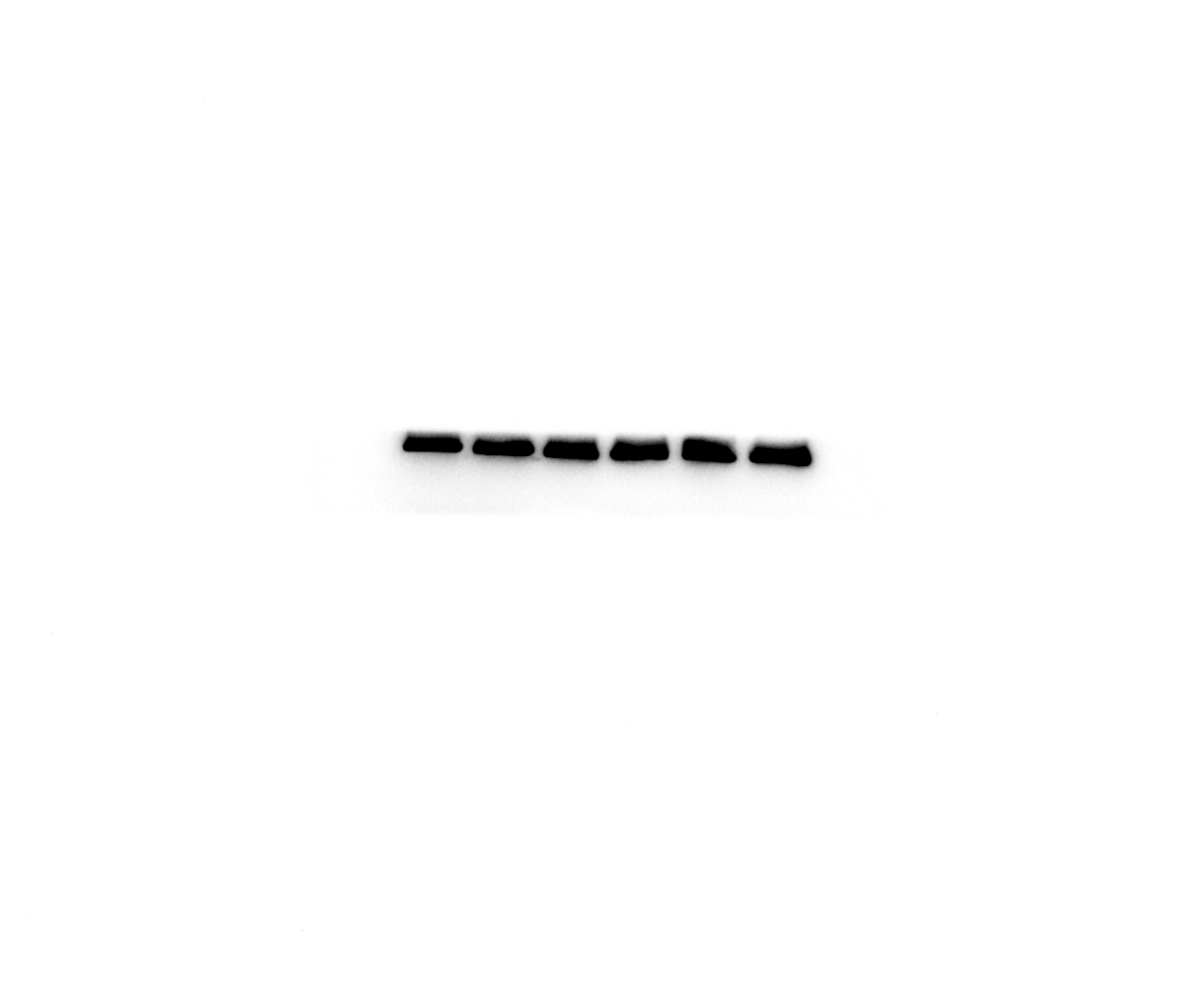

Supplement: Supplementary file 5 [file DataSheet1.zip › Hep3B/MTOR.Tif]

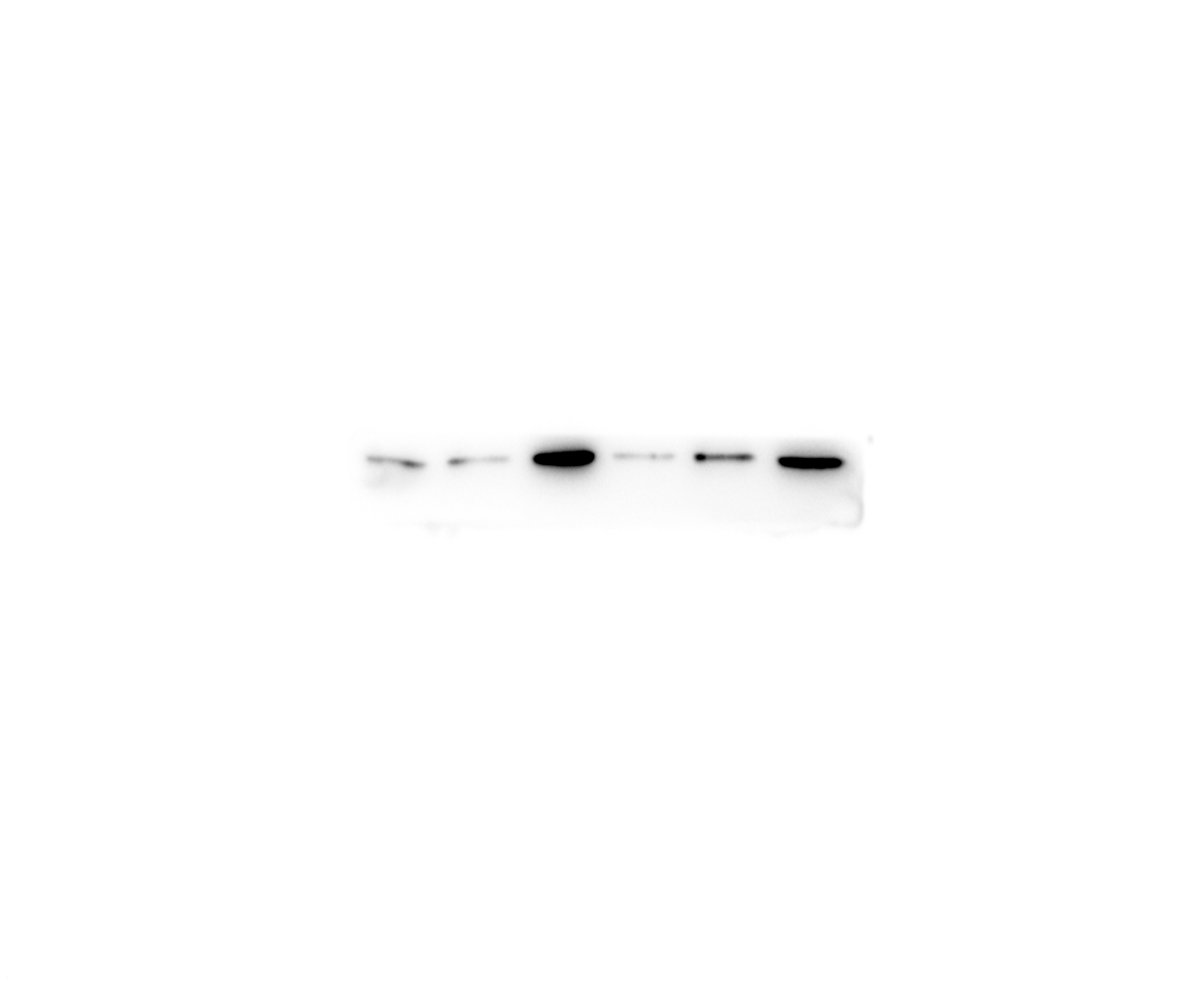

Supplement: Supplementary file 5 [file DataSheet1.zip › Hep3B/P-AKT.Tif]

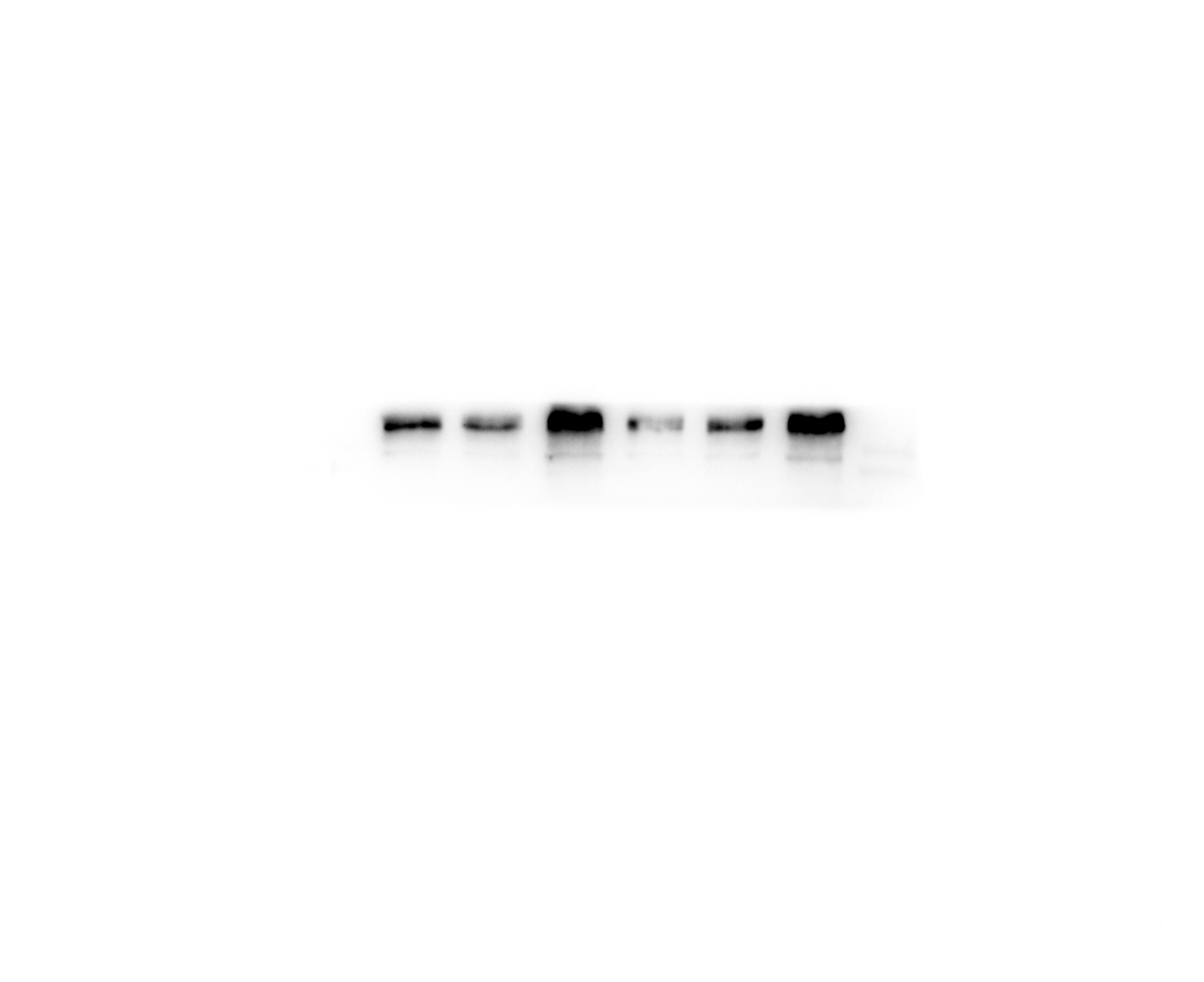

Supplement: Supplementary file 5 [file DataSheet1.zip › Hep3B/p-mtor.Tif]

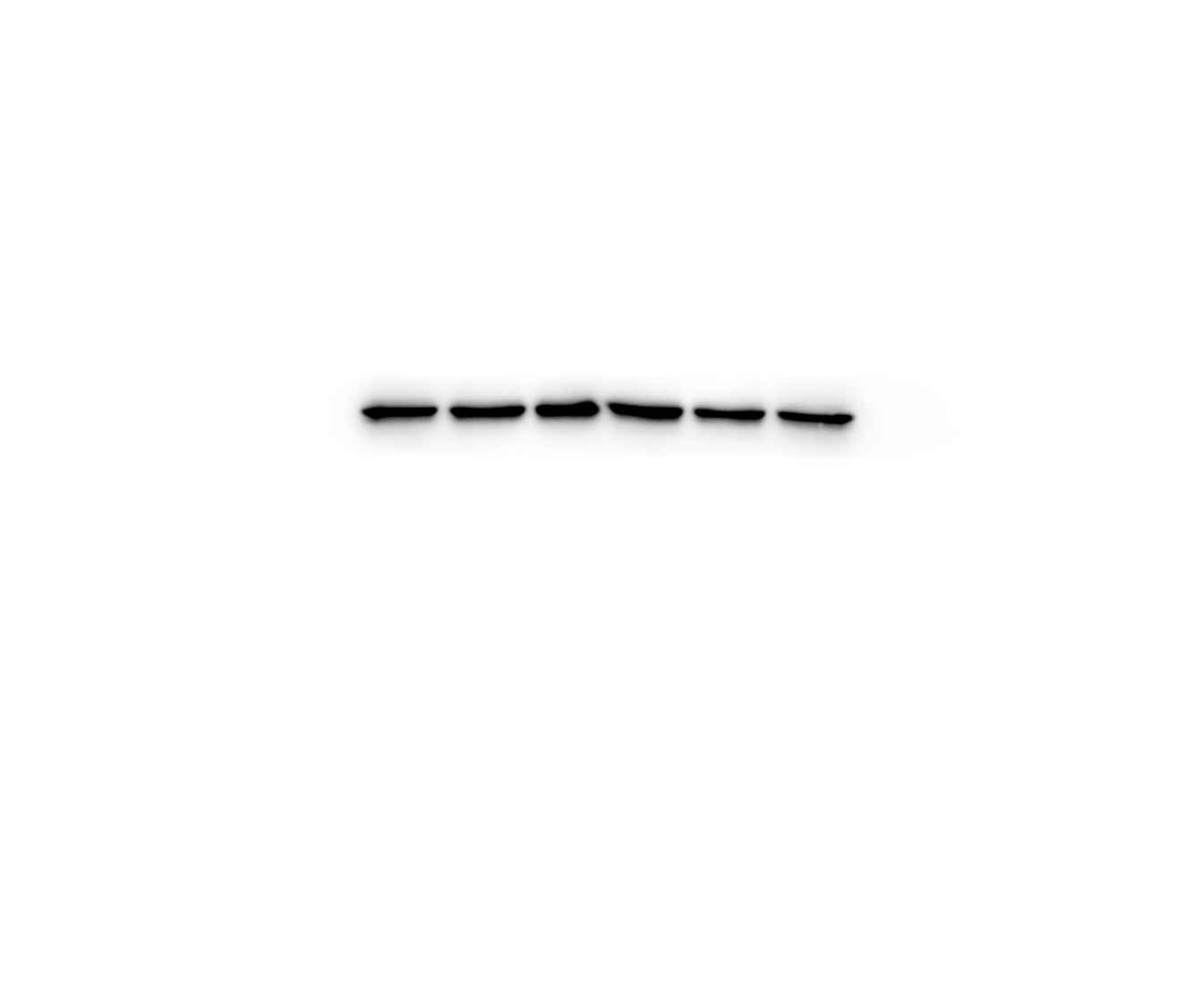

Supplement: Supplementary file 5 [file DataSheet1.zip › Huh7/akt.Tif]

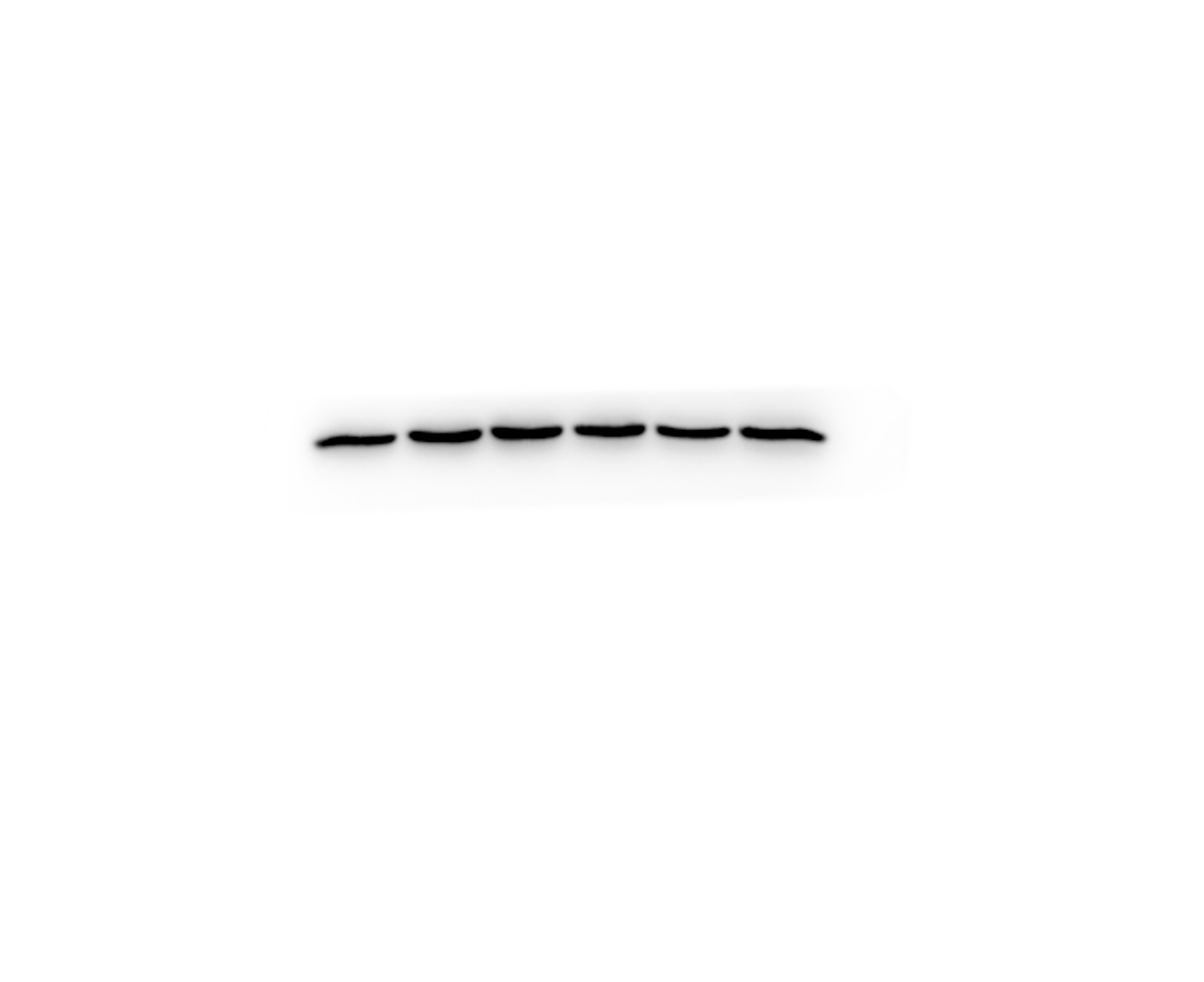

Supplement: Supplementary file 5 [file DataSheet1.zip › Huh7/gapdh.Tif]

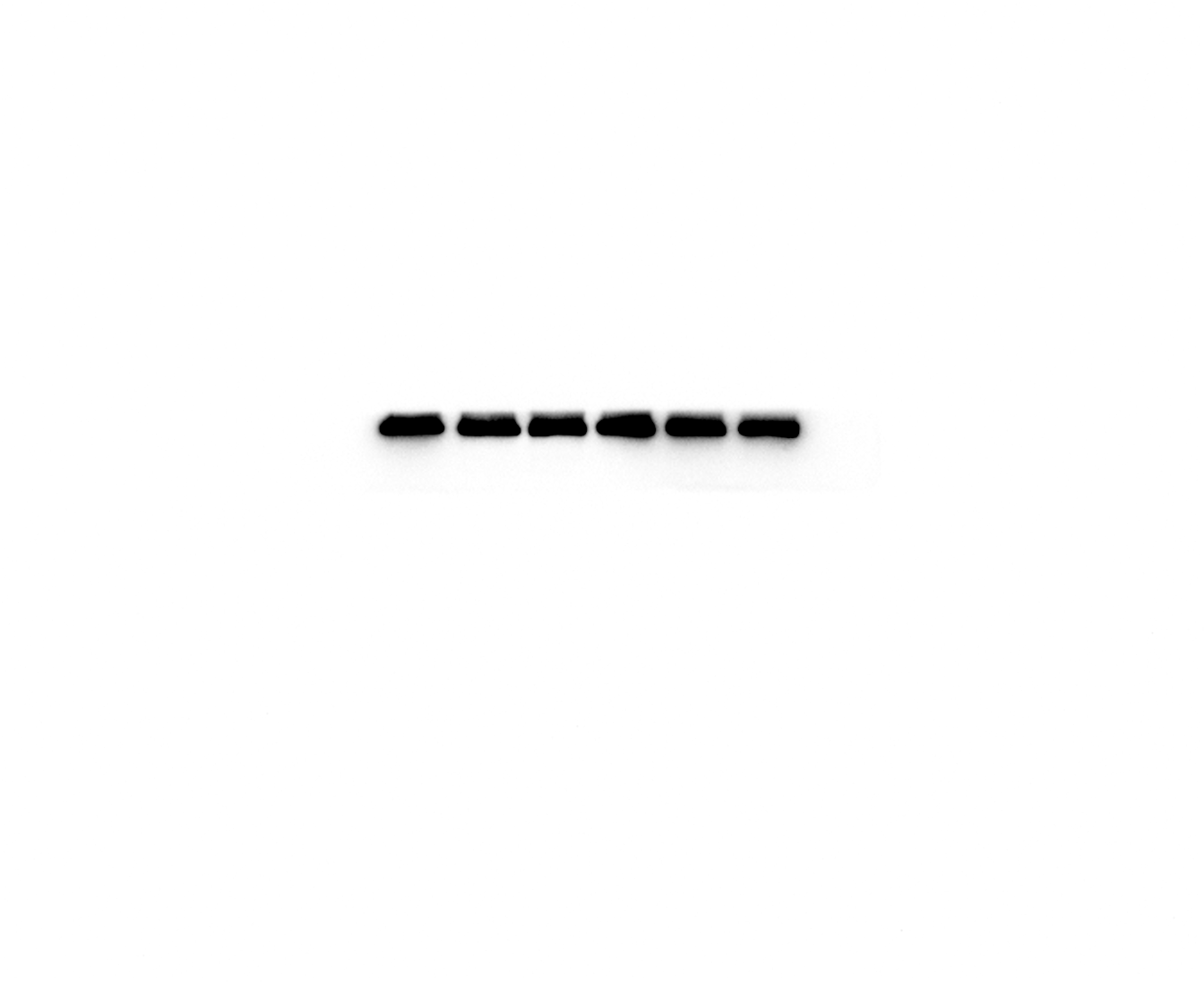

Supplement: Supplementary file 5 [file DataSheet1.zip › Huh7/MTOR.Tif]

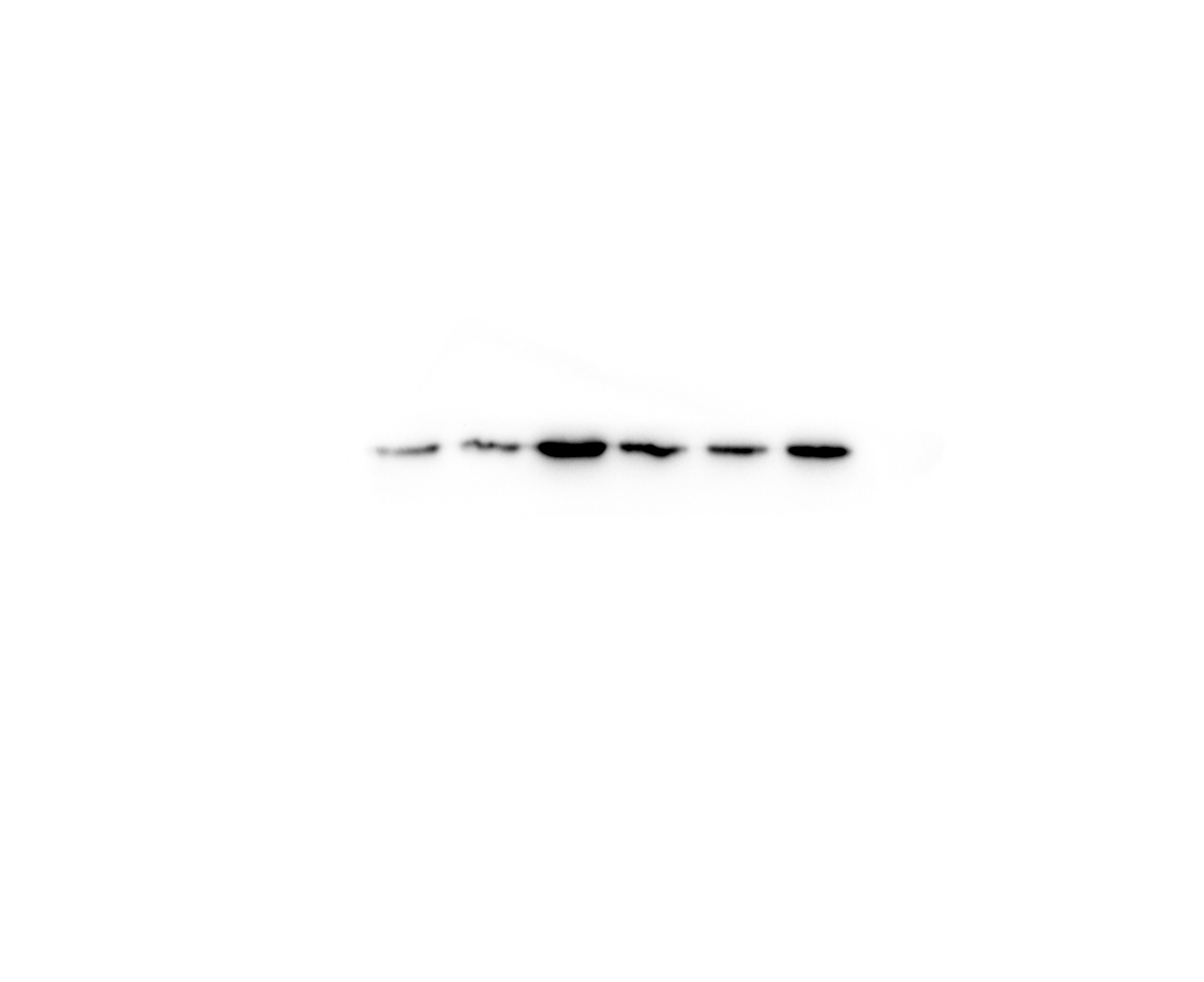

Supplement: Supplementary file 5 [file DataSheet1.zip › Huh7/P-AKT.Tif]

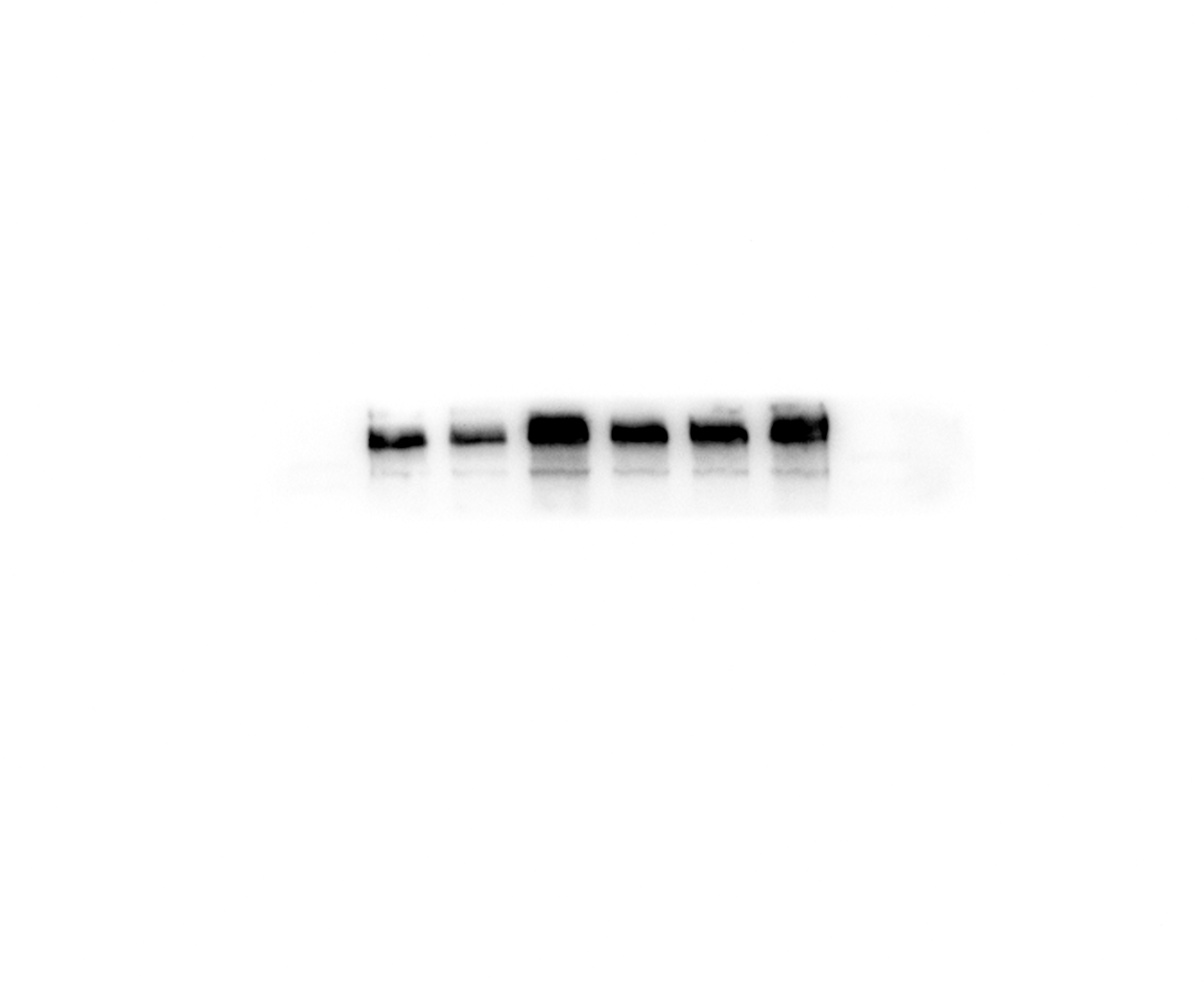

Supplement: Supplementary file 5 [file DataSheet1.zip › Huh7/p-mtor.Tif]

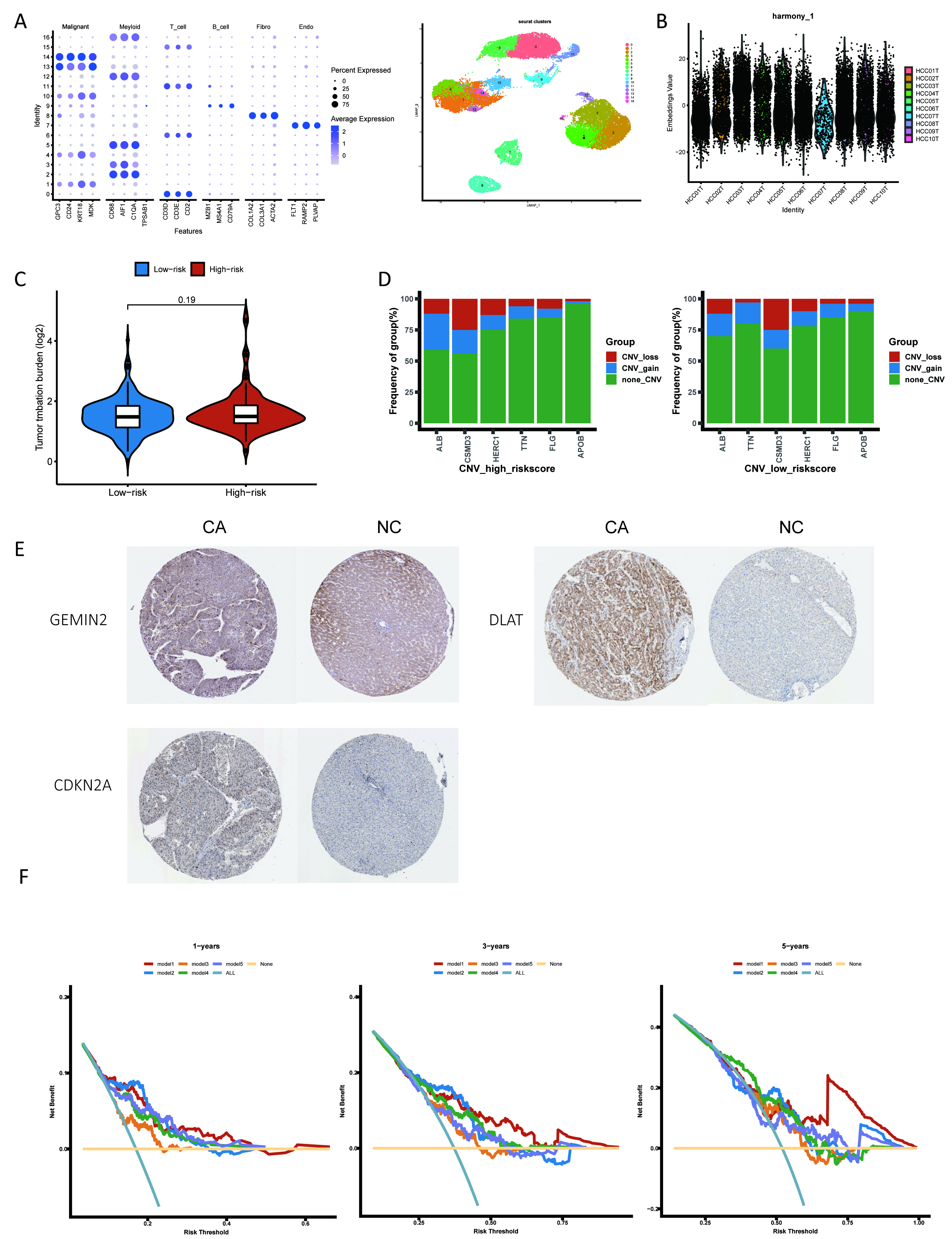

Supplement: Supplementary file 7 [file Image1.TIF]

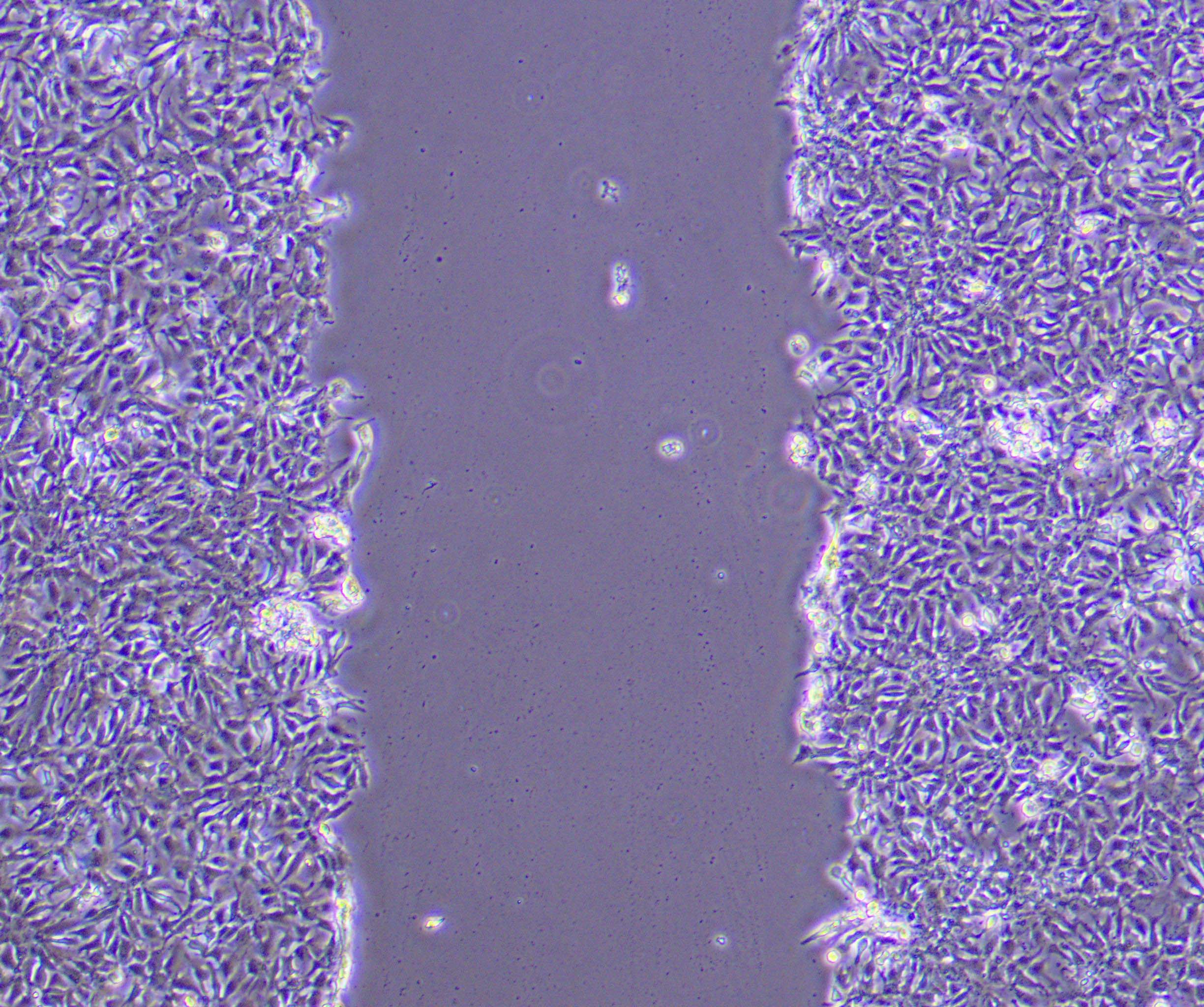

Supplement: Supplementary file 8 [file DataSheet2.ZIP › 0h hep3B NC.jpg]

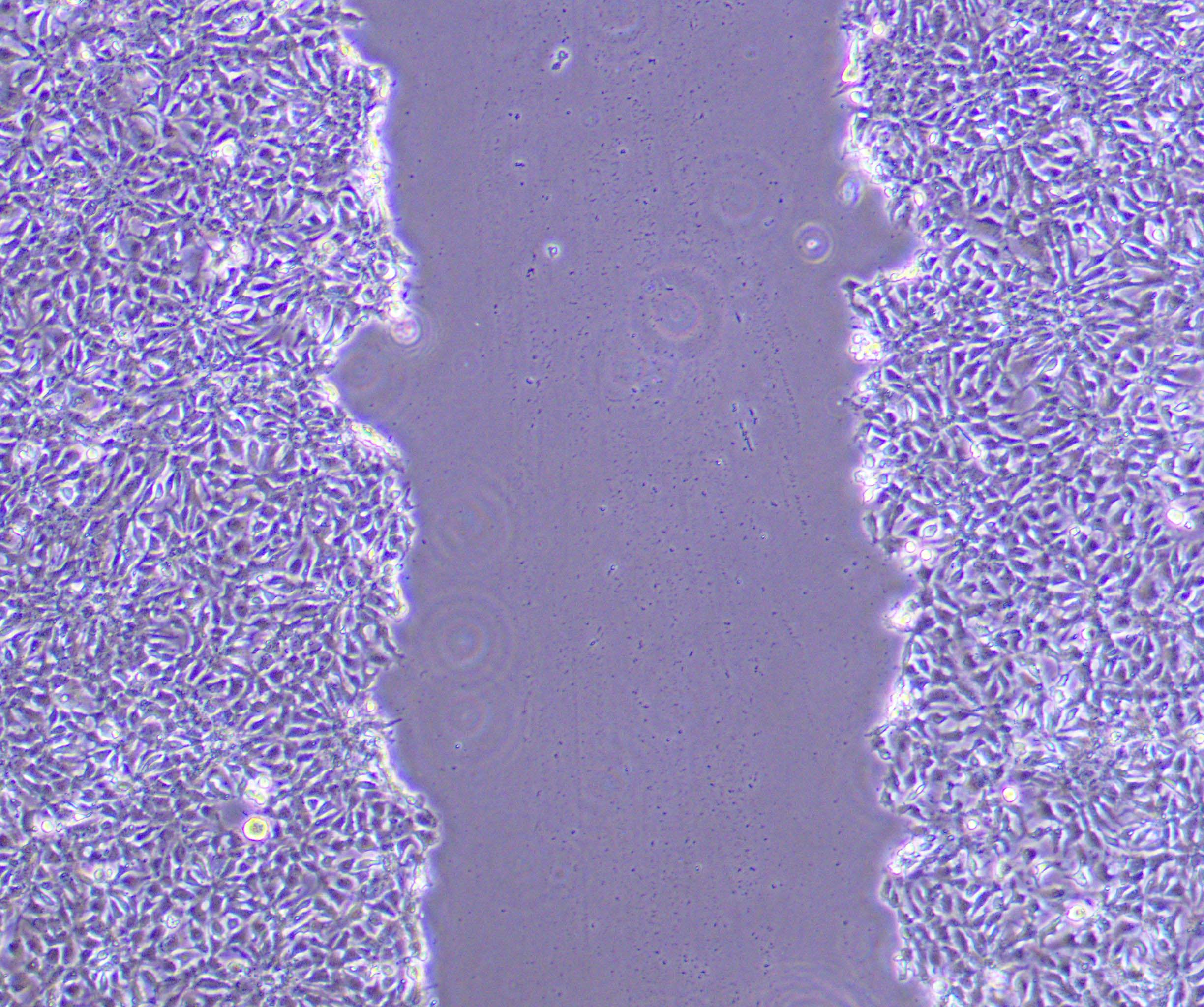

Supplement: Supplementary file 8 [file DataSheet2.ZIP › 0h hep3b Si.jpg]

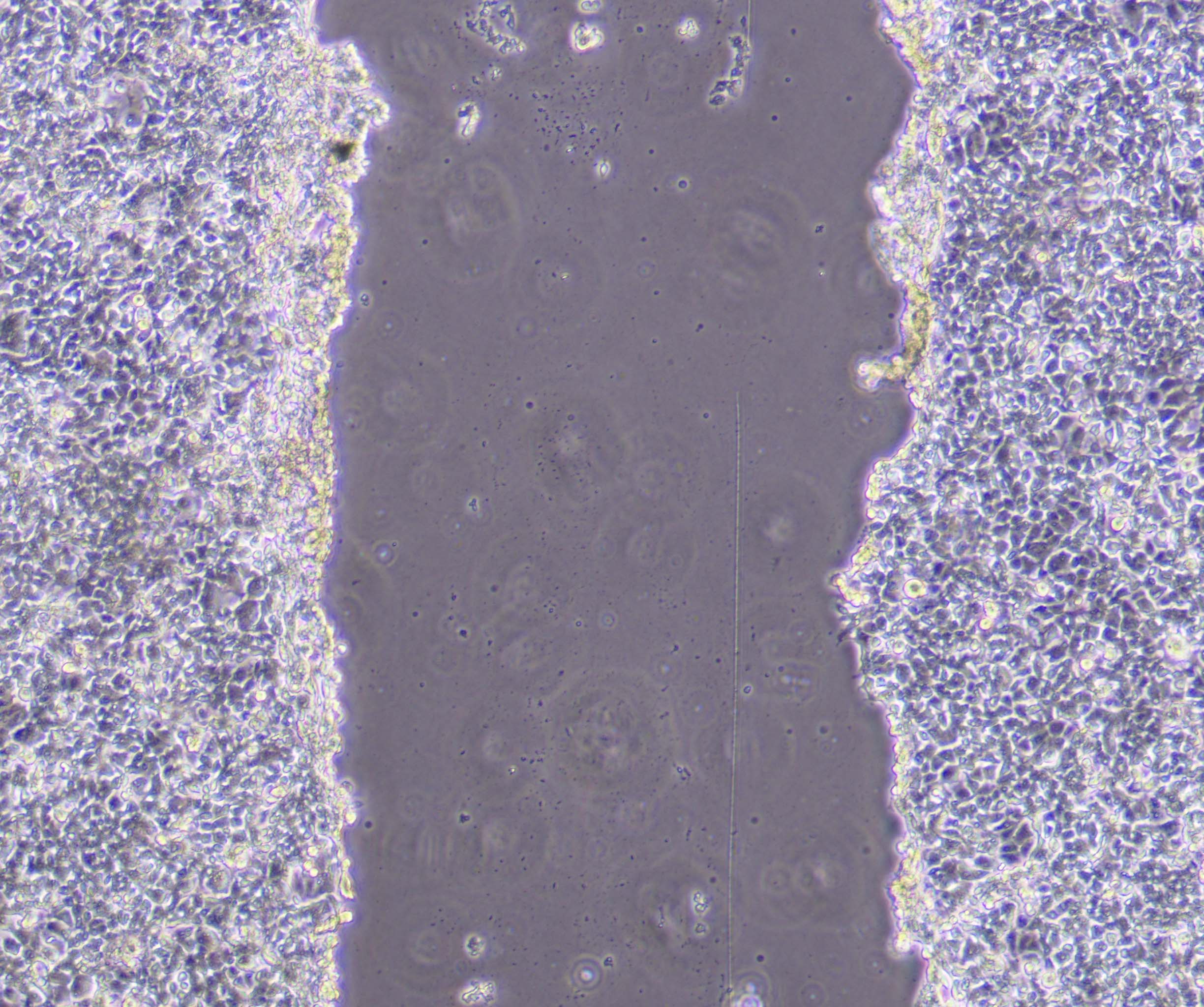

Supplement: Supplementary file 8 [file DataSheet2.ZIP › 0h huh7 NC.jpg]

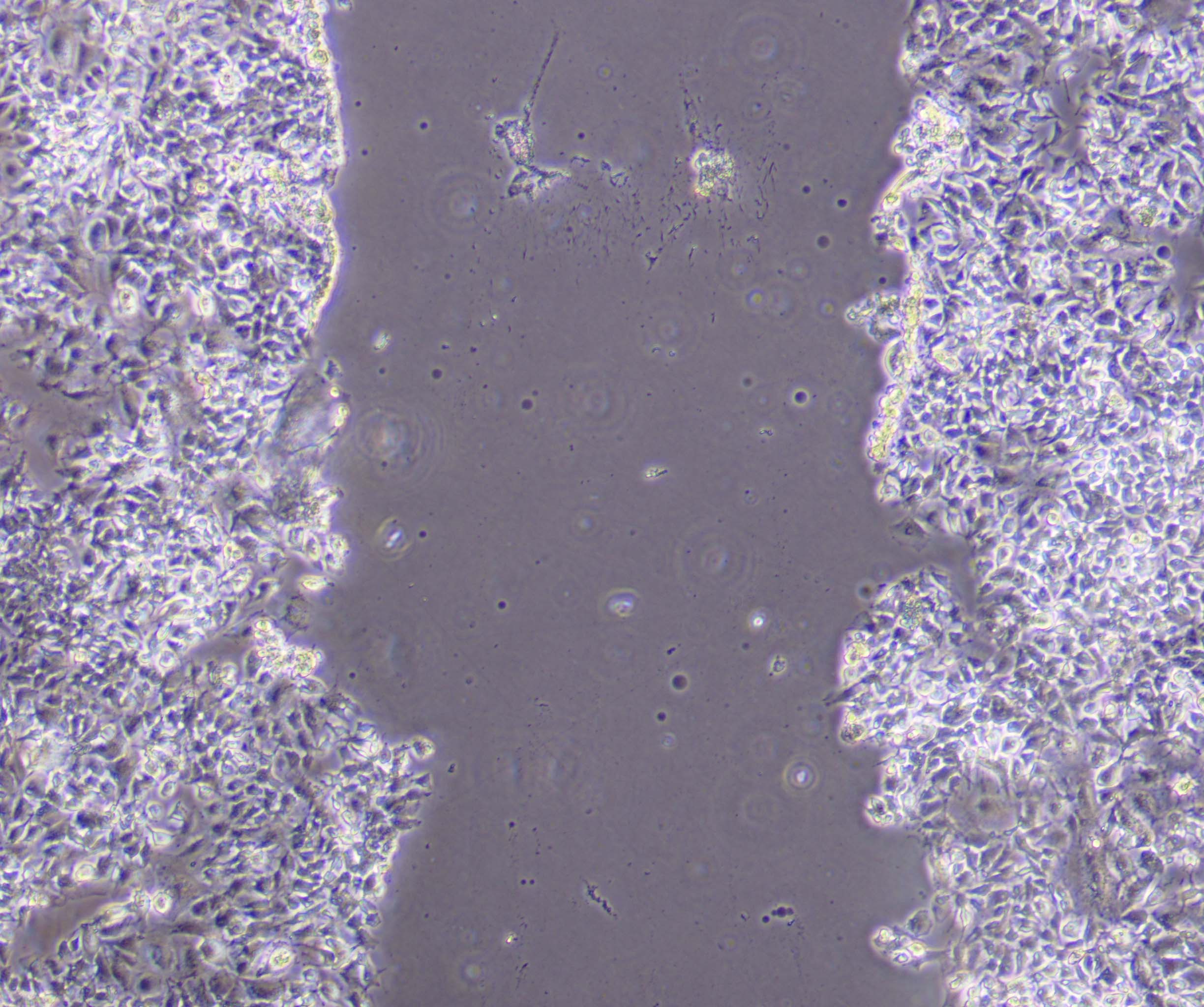

Supplement: Supplementary file 8 [file DataSheet2.ZIP › 0h huh7 Si.jpg]

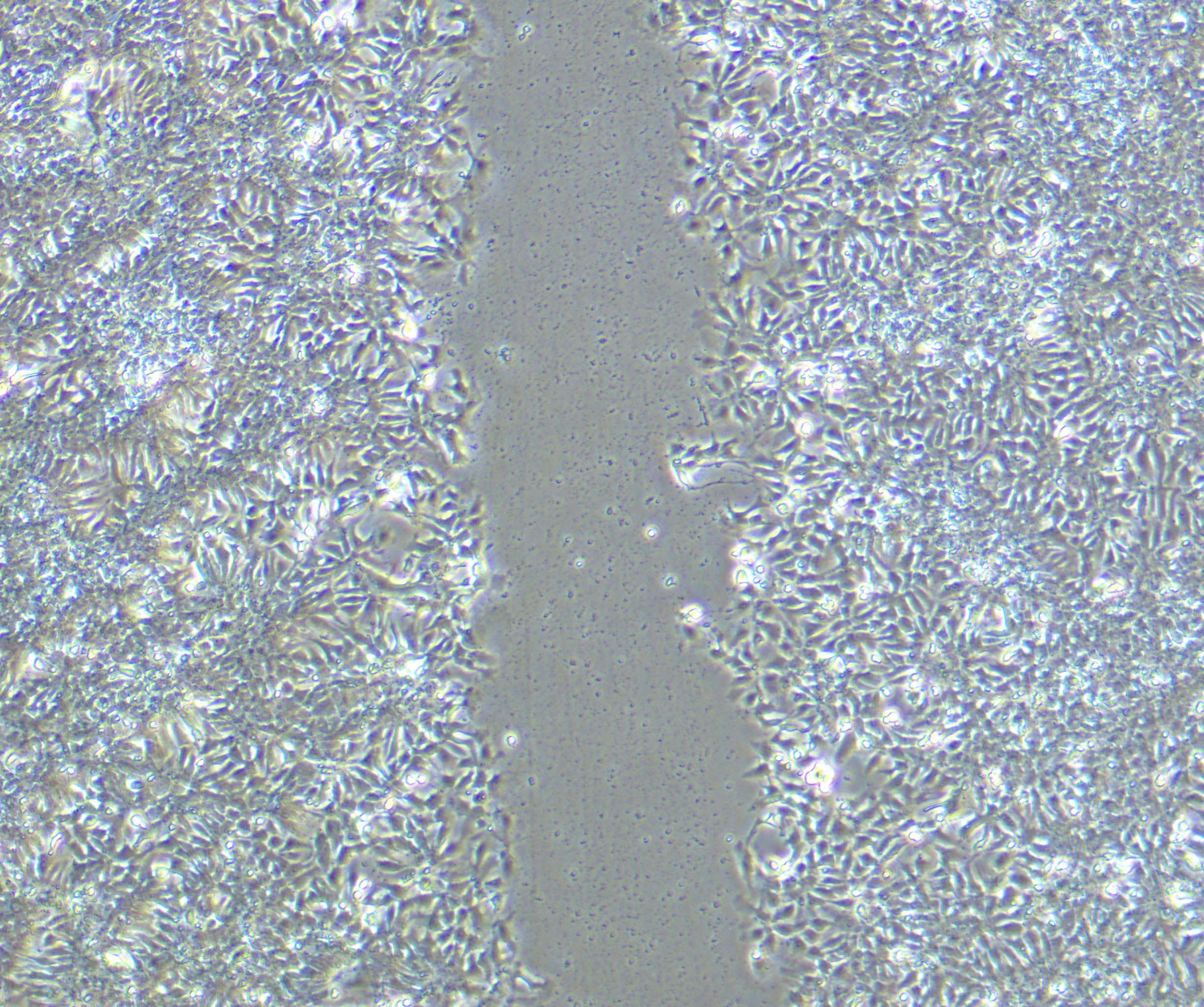

Supplement: Supplementary file 8 [file DataSheet2.ZIP › 30h hep3B NC.jpg]

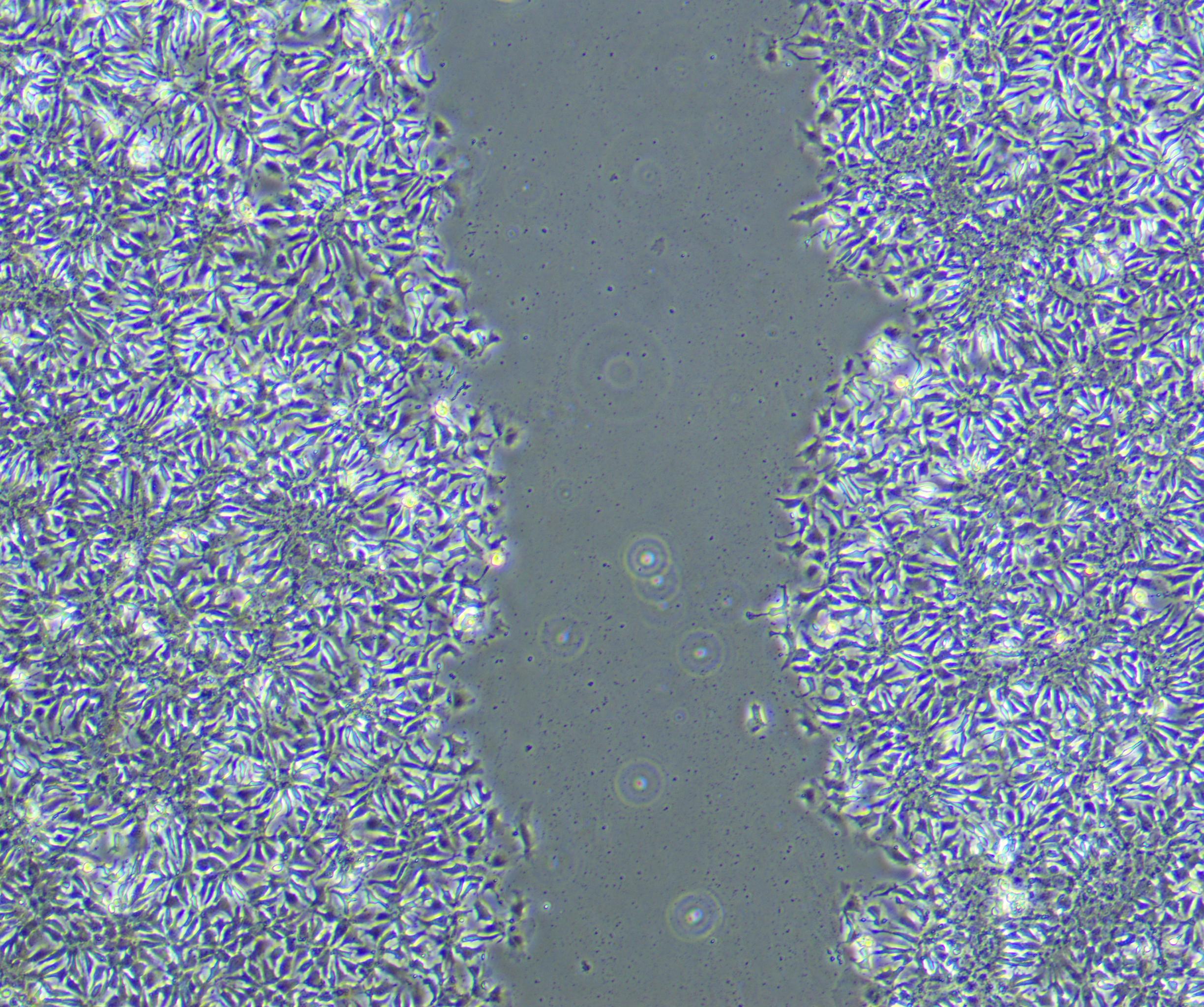

Supplement: Supplementary file 8 [file DataSheet2.ZIP › 30h hep3B Si .jpg]

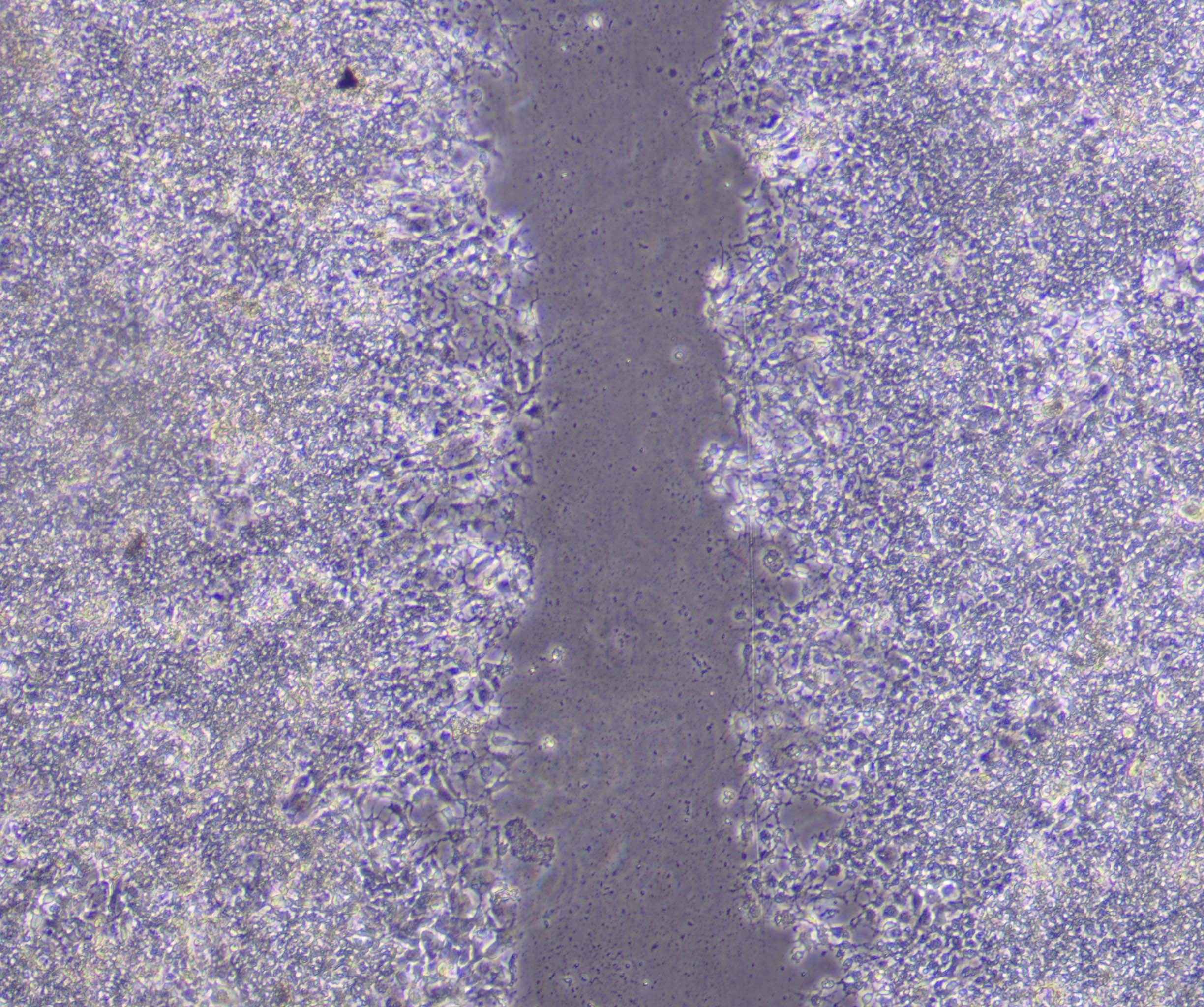

Supplement: Supplementary file 8 [file DataSheet2.ZIP › 30h huh7 NC.jpg]

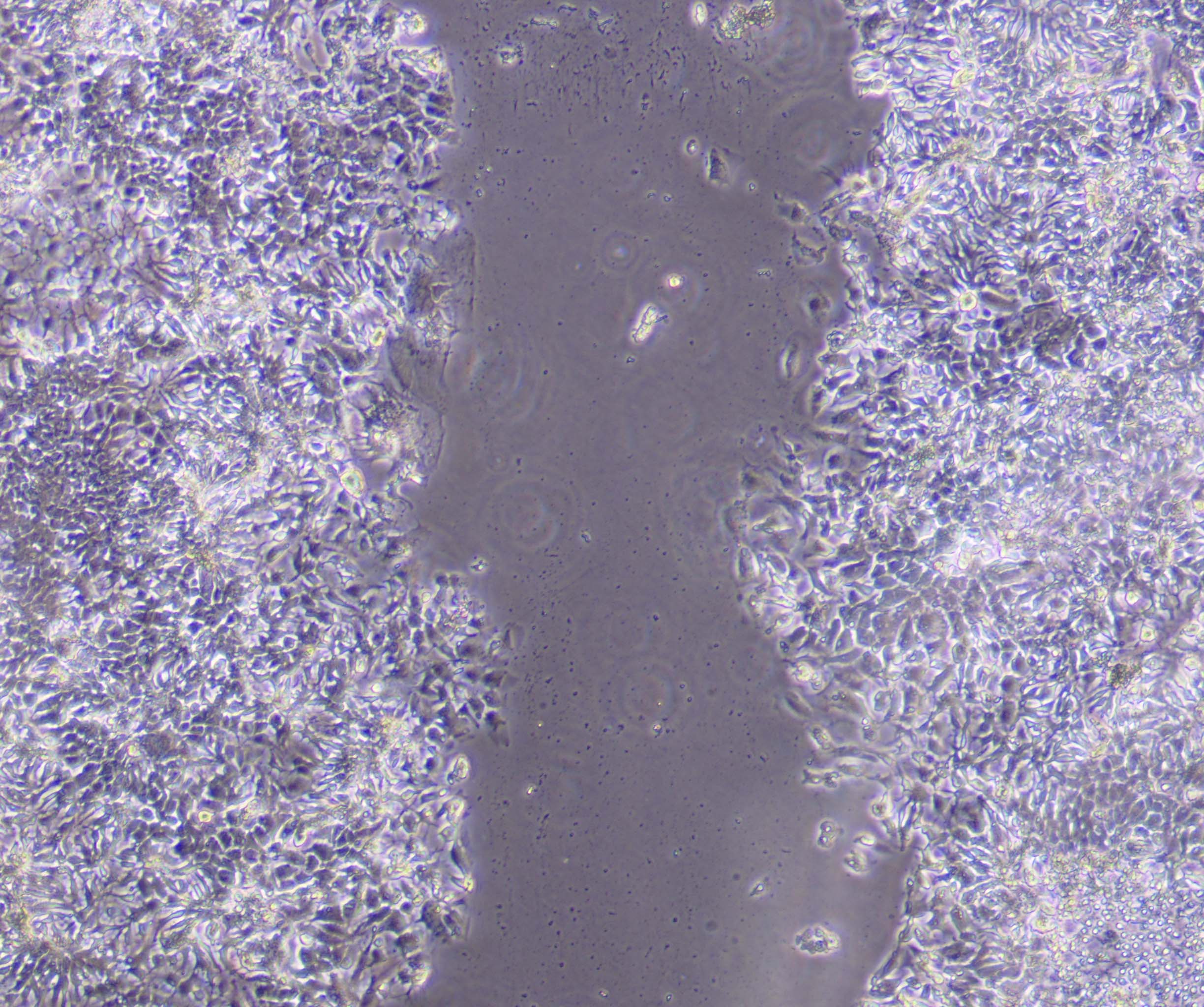

Supplement: Supplementary file 8 [file DataSheet2.ZIP › 30h huh7 Si.jpg]
